# New Frontiers in Cereal and Pseudocereal Germination: Emerging Inducers for Maximizing Bioactive Compounds

**DOI:** 10.3390/foods14173090

**Published:** 2025-09-02

**Authors:** Hans Himbler Minchán-Velayarce, Atma-Sol Bustos, Luz María Paucar-Menacho, Julio Vidaurre-Ruiz, Marcio Schmiele

**Affiliations:** 1 Programa de Doctorado en Ingeniería Agroindustrial mención Transformación Avanzada de Granos y Tubérculos Andinos, Universidad Nacional del Santa, Nuevo Chimbote 02712, Peru; 2024818010@uns.edu.pe; 2 Departamento Académico de Agroindustria y Agronomía, Facultad de Ingeniería, Universidad Nacional del Santa, Chimbote 02712, Peru; abustos@uns.edu.pe (A.-S.B.); luzpaucar@uns.edu.pe (L.M.P.-M.); 3 Centro de Investigación e Innovación en Productos Derivados de Cultivos Andinos CIINCA, Universidad Nacional Agraria La Molina, Lima 15024, Peru; vidaurrejm@lamolina.edu.pe; 4 Institute of Science and Technology, Federal University of Jequitinhonha and Mucuri Valleys (UFVJM), Diamantina 39100-000, Brazil; 5 School of Food Engineering, University of Campinas, Campinas 13083-862, Brazil

**Keywords:** induced germination, phytochemical elicitation, bioactive cereals, GABA enrichment, ultrasound germination, UV-B seed radiation, agricultural cold plasma, concurrent fermentation, functional pseudocereals, phenolics bioavailability

## Abstract

This systematic review analyzes emerging inducers that optimize the germination process of cereals and pseudocereals to enhance bioactive compound production, categorizing them as physical (UV-B radiation, electromagnetic fields, ultrasound, cold plasma), chemical (phytohormones, minerals, growth regulators), and biological (concurrent fermentation, microbial extracts). The results reveal that these inducers significantly increase specific metabolites such as GABA enrichment (up to 800%), phenolic compounds (50–450%), and carotenoids (30–120%) in various bioactive cereals and functional pseudocereals. The underlying mechanisms include enzymatic activation, signal transduction, and controlled stress responses, which improve the bioavailability of phenolics and other bioactive compounds. Critical technological considerations for industrial implementation, bioavailability, and biological efficacy of these compounds are addressed. Synergies between inducers demonstrate exceptional potential for developing ingredients with optimized bioactive properties, especially when combining physical and biological processes. This integrated approach represents a promising frontier in food technology for producing cereals and pseudocereals with enhanced nutritional and functional profiles, applicable in chronic disease prevention and functional food formulation.

## 1. Introduction

Cereals and pseudocereals are fundamental components of global human nutrition, representing a significant proportion of caloric intake and contributing substantially to protein supply, particularly in developing countries [1,2,3,4]. These grains have undergone various technological processes for decades to improve their nutritional, functional, and sensory properties. However, controlled germination has emerged as a promising biotechnology to enhance their nutritional and functional value through systematic activation of endogenous metabolic pathways [5,6,7,8,9].

Germination involves the transition of seeds from dormancy to metabolic activity through a complex physiological process that triggers profound biochemical transformations. These transformations include macronutrient hydrolysis, de novo synthesis of bioactive compounds, and enzymatic modulation [10,11,12,13]. The process induces significant changes in phytochemical profiles [7,14], enhancing the concentration and bioavailability of bioactive compounds with functional properties such as *γ*-aminobutyric acid (GABA) [5,9,15,16,17], phenolic compounds [8,14,18], vitamins [19,20], bioavailable minerals [21,22,23], and antioxidant enzymes [16,24,25].

Regular consumption of these foods has been correlated to health benefits [7,26,27,28] such as reduced cardiovascular risk [7,26], improved glycemic control [15,21], inflammatory modulation [14], and immune enhancement [10,11]. These effects result from the synergistic action of bioactive compounds [8,27,29]. Recent studies show that the accumulation of bioactive compounds is significantly enhanced by specific inducers (physical, chemical, or biological) [19,30,31]. These inducers modulate metabolic pathways [9,31,32] and activate controlled stress responses [31,33], resulting in increased synthesis and accumulation of secondary metabolites [14,34,35].

Physical inducers include electromagnetic radiation (UV light, magnetic fields) and mechanical treatments (ultrasound), and emerging technologies such as cold plasmas and pulsed electric fields stand out [6,15,31,36,37]. Chemical inducers include phytohormones, plant-derived elicitors, specific minerals, and growth regulators [38,39,40], while biological inducers comprise concurrent fermentation processes and application of microbial extracts [5,24,41,42]. Each category of inducers acts through specific molecular mechanisms to trigger adaptive responses that lead to the accumulation of bioactive compounds [17,32,43,44].

The technological implementation of these inducers requires a comprehensive understanding of their specific effects, optimal application conditions, potential synergies, and technical limitations, especially considering the variability in response according to species, cultivar, and environmental conditions [19,45,46,47]. Evaluating the bioavailability and biological efficacy of enhanced bioactive compounds is fundamental for determining their nutritional and functional relevance [30,38,48,49].

Developing functional foods from germinated cereals and pseudocereals under controlled conditions with application of specific inducers emerged a promising strategy to address contemporary nutritional challenges. This approach aligns with global trends towards minimally processed foods with demonstrable functional properties [50,51,52,53]. However, the transition from experimental applications to industrial implementations presents multiple technological, regulatory, and commercial challenges requiring systematic research approaches [2,28,43,44].

Previous reviews have addressed germination-induced bioactive enhancement in cereals and pseudocereals, yet this systematic review distinguishes itself through three unique contributions. First, it integrates bibliometric analysis with VOSviewer to identify emerging research trends and knowledge gaps in post-2020 research. Second, it provides the first comprehensive classification system for emerging inducers, systematically categorizing physical, chemical, and biological approaches with their specific mechanisms of action. Third, unlike previous reviews focusing on individual treatments, this work emphasizes synergistic combinations of inducers and provides quantitative comparisons of bioactive compound enhancement across different matrices.

This review comprehensively analyzes emerging inducers for cereal and pseudocereal germination, examining their mechanisms of action, effectiveness in enhancing specific bioactive compounds, technological considerations for industrial implementation, bioavailability and biological efficacy of enhanced compounds, applications in functional food development, and future research directions. This critical synthesis establishes a conceptual and technical framework facilitating knowledge translation from basic research to industrial and nutritional applications with significant public health impact potential.

## 2. Search Strategies and Brief Bibliometric Analysis

The present study was designed under a systematic bibliometric approach to identify, select, and analyze the most relevant scientific literature on emerging inducers for the germination of cereals and pseudocereals, with emphasis on the enhancement of bioactive compounds. This methodology enables comprehensive mapping of current research trends while identifying knowledge gaps and emerging opportunities in the field.

Search Strategy. The bibliographic search was conducted in three international databases: Scopus, Web of Science, and Science Direct, considering publications between 2015 and 2025. Structured combinations of Boolean terms were used to cover the different dimensions of the study topic. The search equations included the following:

(TITLE-ABS-KEY(((“germination” OR “sprouting”) AND (“time” OR “duration”) AND (“temperature”) AND (“cereals” OR “pseudo-cereals”) AND (“bioactive compounds” OR “antioxidants” OR “phenolics” OR “nutritional improvement”)) AND ALL (“temperature” OR “time”)) AND PUBYEAR > 2015 AND PUBYEAR < 2026).

Additional targeted searches focused on specific crop species and physical treatments to ensure comprehensive coverage of emerging inducer technologies. These supplementary searches captured literature on physical treatments, electromagnetic applications, and novel enhancement methodologies not covered by general search terms.

Study Selection and Analysis. The search strategy initially identified 440 articles, which were subjected to a screening process to evaluate their relevance according to the inclusion criteria. Finally, 126 articles were selected for their direct relationship with the research focus and objectives. Bibliographic management was performed using Zotero 7.0.15, which allowed the extraction of metadata in JSON format for subsequent analysis.

Inclusion Criteria. Publication type: original articles, peer-reviewed, published in indexed scientific journals. Thematic focus: studies evaluating controlled germination processes in cereals and pseudocereals, including main species such as *Chenopodium quinoa*, *Chenopodium pallidicaule*, *Amaranthus* spp., *Panicum miliaceum*, *Setaria italica*, *Triticum aestivum*, *Hordeum vulgare*, *Zea mays*, *Oryza sativa*, and *Fagopyrum esculentum*. Inducer application: studies investigating physical, chemical, or biological inducer applications during germination, such as UV light, ultrasound, electromagnetic fields, phytohormones, minerals, microbial extracts, or concurrent fermentation. Bioactive compound evaluation: studies reporting quantitative data on bioactive compound presence or increases, including TPC, flavonoids, GABA, carotenoids, vitamins, bioactive peptides, and antioxidant enzymes. Experimental design: research with clearly defined germination parameters (time, temperature, relative humidity, photoperiod, treatment type). Temporal coverage: publications from January 2015 to January 2025 with full text access. Language: English publications.

Exclusion Criteria. Narrative reviews, brief communications, editorials, book chapters, and work without peer review. Studies that did not use germination as a central treatment or that applied inducers in later stages (e.g., drying, cooking, or extrusion). Research focused exclusively on digestibility, starch, proteins, agronomic profile, or plant development, without evaluating bioactive compounds. Studies reporting only qualitative or descriptive results, without verifiable numerical data. Preprints, duplicate articles, or without full text access.

Brief Bibliometric Analysis. VOSviewer 1.6.20 was employed for comprehensive bibliometric analysis, enabling construction and visualization of co-occurrence networks of terms with quantitative relationship mapping. A minimum threshold of five occurrences was established for each term, resulting in 76 terms selected for analysis with sufficient statistical significance. The network visualization incorporated three complementary approaches: network visualization showing term relationships, overlay visualization incorporating temporal dimensions, and density visualization reflecting connection intensity.

**Figure 1:**
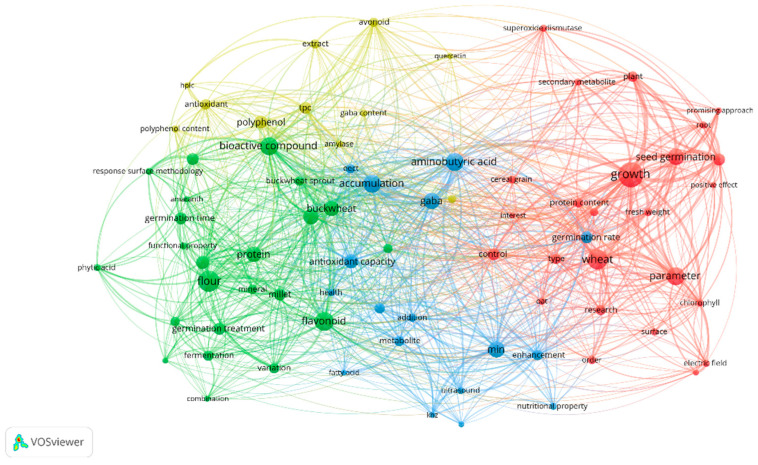
Co-occurrence network of research terms in research on emerging inducers for the germination of cereals and pseudocereals showing four thematic clusters: red (plant growth/germination), green (cereals/nutrition), blue (inducers/metabolism), and yellow (bioactive compounds). Line thickness indicates co-occurrence strength; node size reflects term frequency in analyzed literature.

Bibliometric Network Structure. The resulting bibliometric network (Figure 1) revealed a clear organization in four main clusters: Cluster 1 (25 terms): Centered on aspects of plant growth and seed germination, with predominant terms such as “growth” (146 links), “wheat” (109 links), and “seed germination” (92 links). The weighted average publication year was 2022. Cluster 2 (23 terms): Focused on specific cereals/pseudocereals and their nutritional properties, highlighting “bioactive compound” (96 links), “flavonoid” (96 links), and “flour” (94 links). The weighted average year was 2022.13. Cluster 3 (17 terms): Related to inducers and metabolic processes, with main terms such as “aminobutyric acid” (115 links), “accumulation” (101 links), and “GABA” (97 links). The weighted average year was 2021. Cluster 4 (11 terms): Concentrated on specific bioactive compounds and antioxidant capacity, highlighting “polyphenol” (73 links), “flavonoid” (61 links), and “TPC” (60 links). The weighted average year was 2020.

Bridge terms connecting multiple clusters were identified as strategic research convergence points, including “growth”, “aminobutyric acid”, “wheat”, “accumulation”, and “GABA”, all demonstrating connections across the four main clusters. These terms indicate conceptual convergence points and suggest opportunities for interdisciplinary research approaches integrating multiple thematic areas.

**Figure 2:**
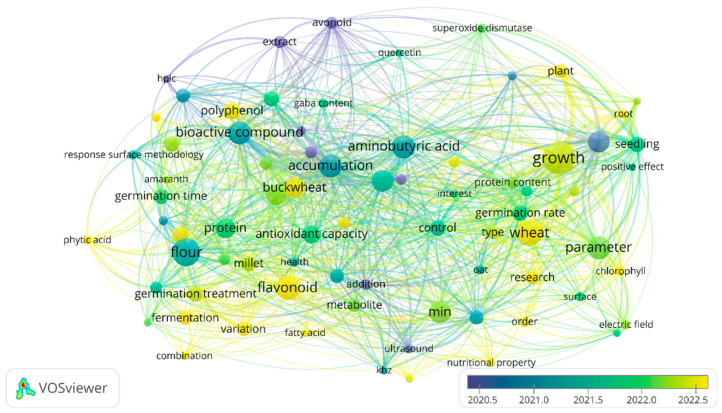
Temporal overlay visualization of the co-occurrence network of terms in research on emerging inducers for the germination of cereals and pseudocereals. Color gradient represents publication timeline: blue/cool colors indicate older research (2020–2021), while yellow/warm colors represent recent research (2022–2023).

Temporal Trends. The chronological analysis (Figure 2) revealed an evolution in research approaches. The most recent terms (2022–2023) were concentrated in “combination” (2023.4), “polyphenol content” (2023.33), “flavonoid” (2023.06), and “corn” (2023.00), indicating a growing interest in specific compounds and treatment combinations. The more established terms (2018–2020) included “HPLC” (2018.00), “amylase” (2018.83), and “extract” (2019.63), mainly associated with analytical methodologies.

Specific Terms of Interest. Pseudocereals emerged as prominent study models, with particular attention to “buckwheat” (87 links, year 2022.46), “quinoa” (92 links, year 2022.15), and “amaranth” (34 links, year 2022.20). These terms demonstrated strong associations with bioactive compounds and functional properties, suggesting their increasing recognition as superior matrices for bioactive enhancement applications.

Among emerging inducers, “electric field” (33 links, year 2022.17), “ultrasound” (53 links, year 2020.67), “fermentation” (48 links, year 2022.56), and “germination treatment” (58 links, year 2022.30) showed significant research presence. Analysis of connectivity patterns revealed strong associations with specific bioactive compounds, particularly GABA and flavonoids, indicating successful mechanistic understanding and practical application development.

This bibliometric analysis provides a structured overview of current research on emerging inducers for cereal and pseudocereal germination, identifying research concentration areas, emerging trends, and future study opportunities. Term convergence between clusters suggests an interdisciplinary field with multiple methodological and conceptual approaches, while temporal trends indicate evolution toward practical applications and synergistic enhancement strategies.

## 3. Fundamentals of Germination and Its Impact on Bioactive Compounds

### 3.1. Germination Process: Physiological and Biochemical Aspects

The germination process can be divided into three main phases: (1) Imbibition Phase: dry seeds rapidly absorb water, activating cellular metabolism and triggering biochemical changes [54,55]; (2) The Metabolic Activation Phase: water absorption slows while key metabolic processes activate, including synthesis of hydrolytic enzymes that degrade seed reserve compounds [52]; (3) The Visible Growth Phase: water absorption resumes, leading to radicle emergence and seedling establishment [54,55].

During these phases, profound physiological and biochemical changes occur that fundamentally alter seed composition and nutritional properties [54,56,57]. Enzymatic pathways activate, reserve compounds degrade, new proteins and secondary metabolites synthesize, and structural reorganization of seed tissues takes place [58,59,60].

Various environmental factors can negatively affect the germination process, including water stress, salinity, and heavy metal presence, which alter seed physiological and biochemical parameters through disruption of normal metabolic processes [58,59,61,62,63]. Conversely, treatments such as seed conditioning (priming) can improve plant stress tolerance and promote more efficient germination [58,59,60]. Understanding these mechanisms is fundamental for optimizing germination and maximizing bioactive compound composition.

### 3.2. Main Bioactive Compounds in Germinated Seeds

Bioactive compounds represent functional components present in grains that gain enhanced relevance during germination, promoting human health benefits beyond basic nutritional requirements [26,27]. Germination systematically modifies the phytochemical profile through activation of biosynthetic pathways and release of bound compounds, generating a diverse spectrum of functional metabolites with documented health benefits [14,18,21,26]. The main bioactive groups identified in germinated seeds include phenolic compounds (TPC) [1,5,8,14,16,18,23,25,42,50,52,53,64,65], GABA [3,5,11,16,17,25,26,66,67], bioactive peptides (BIOP) and free amino acids (FAA) [16,17,23,26,30,31,41,50,68,69,70,71], vitamins [19,20,72,73], bioavailable minerals [19,21,23,26,28,50], antioxidant enzymes [74,75], specialized phytochemicals such as CAROTS and avenanthramides [11,20,26,35], and dietary fiber fractions [1,16,21,23,26,53,74]. Table 1 provides a classification to understand their diversity and supports the analysis of their functional properties and mechanisms of action.

#### 3.2.1. Phenolic Compounds

Phenolic compounds constitute the most important and widely studied phytochemical group in germinated seeds, serving as primary contributors to antioxidant capacity and functional properties. Research has documented total phenols, total flavonoids, and total 3-deoxy-anthocyanidins in germinated red sorghum (*Sorghum bicolor*) and pearl millet (*Pennisetum glaucum*) [24]. Studies also identified polyphenols and total flavonoids in germinated foxtail millet (*Setaria italica* L.) [42]. Researchers characterized detailed phenolic compound profiles in germinated buckwheat (*Fagopyrum esculentum*) and quinoa (*Chenopodium quinoa*), distinguishing between flavonoids (rutin, quercetin, kaempferol, chrysin, hesperidin, catechin, epicatechin) and phenolic acids (*p*-hydroxybenzoic, chlorogenic, ellagic, salicylic, *p*-coumaric, gentisic, ferulic) [8].

The scientific evidence is considered “moderate” because in vitro studies were presented, but in vivo validation or commercial applications were limited (Table 1). Table 1 systematically categorizes the main bioactive compound groups found in germinated cereals and pseudocereals, detailing their characteristics, health benefits, mechanisms of biological action, and the current level of scientific evidence supporting their functional properties.

Studies identified and quantified various phenolic acids in germinated black rice (*Oryza sativa*), including gallic acid, chlorogenic acid, ellagic acid, ferulic acid, hydroxybenzoic acid, isoferulic acid, *p*-coumaric acid, protocatechuic acid, sinapic acid, and vanillic acid [82]. The same authors detected flavonoids (kaempferol, quercetin, rutin) and anthocyanins (cyanidin-3-galactoside, cyanidin-3-glucoside, cyanidin-3,5-diglucoside, cyanidin-3-rutinoside, malvidin-3-galactoside) in this species. Research reported total phenols, total flavonoids, and specific phenolic compounds (chlorogenic acid, catechin, 4-hydroxybenzoic acid, sinapic acid, rutin, naringin, quercetin, caffeic acid, *p*-coumaric acid, epicatechin) in germinated wheat [32]. Studies evaluated TPC and anthocyanins in germinated blue corn (*Zea mays* L.) and identified TPC in germinated naked barley [5]. Research reported total flavonoids in soft wheat varieties Zauralochka and Erythrosperium, as well as in barley variety Chelyabinets 1 [3].

#### 3.2.2. GABA

GABA’s centrality in co-occurrence network analysis (97 links, Cluster 3) reflects its fundamental role as a bridge compound connecting inducer mechanisms with bioactive enhancement outcomes (Figure 1). As identified in the bibliometric analysis, GABA serves as a key connector between different research clusters, particularly linking metabolic processes (Cluster 3) with plant growth mechanisms (Cluster 1). This cross-cluster connectivity suggests that GABA enhancement strategies may provide synergistic pathways for simultaneously optimizing multiple bioactive profiles during germination.

Germination intrinsically increases GABA content by activating glutamate decarboxylase (GAD), the key enzyme [3,5,10,15,27,66,67,86]. However, external stimuli modulation can further amplify this accumulation.

Physical inducers: Ultrasonication (US) has emerged as a promising technology, and has been shown to increase GABA content in naked barley [66], germinated brown rice [15,31,66,87], germinated wheat [10], and soybean sprouts [10,31], as well as oats [3,11]. This effect is attributed to the activation of GAD and the alteration of cell membrane permeability, which facilitates the absorption of precursors and/or inhibits GABA catabolism [15,15,17,31]. Pulsed light (PL) has also been shown to increase GABA in sprouted corn by promoting the interaction of glutamate with GAD and by modifying cell membrane permeability [9]. Similarly, cold plasma (CAPP), as a physical stimulus, increases GABA content and GAD activity in barley [70] and wheat sprouts [3], which is linked to the synthesis of reactive nitrogen and oxygen species that promote germination and lytic activity [45,88]. UV-B radiation also increases GABA content in blue corn and highland barley sprouts by stimulating GAD activity and the expression of genes related to its synthesis. However, excessive exposure can inhibit this accumulation [26,73,73].

Chemical inducers: Salinity (NaCl) and calcium chloride (CaCl_2_) are recognized as abiotic stress factors that induce GABA accumulation in various plants as a protective response [30,33,37,73,89]. CaCl_2_ can influence GAD activity through the calmodulin pathway [5,33,33]. Sucrose, as an osmotic inducer, has also been shown to induce GABA increase in buckwheat sprouts [33]. Of relevance is pyridoxal phosphate (PLP), a cofactor of GAD, which significantly enhances GABA synthesis by activating this enzyme and modulating glutamate metabolism [30].

Together, these studies demonstrate that, beyond germination alone, the controlled application of physical-chemical inducers represents an effective strategy for intensifying GABA accumulation in grains, thereby enhancing their functional properties of interest for human health, such as lowering blood pressure and relieving stress [3,5,27,30,67]. This evidence underscores the potential of these technologies for developing GABA-enriched functional foods.

Studies have identified GABA in various germinated grain species, with research detecting notable accumulation in germinated naked barley [5], soft wheat varieties Zauralochka and Erythrosperium, and barley variety Chelyabinets [15]. One study focused exclusively on GABA characterization in germinated brown rice (*Oryza sativa* L., variety Nanjing) [3].

Researchers identified GABA along with other bioactive compounds in germinated djulis, a native pseudocereal from Taiwan, while studies also detected GABA and total free amino acids in germinated wheat, demonstrating the compound’s widespread occurrence across diverse cereal and pseudocereal matrices [37,41]. GABA was also detected in germinated sorghum [76] and in germinated kodo millet (*Paspalum scrobiculatum*) [77]. In germinated coix seed (*Coix lacryma-jobi* L.), GABA, soluble proteins, and free amino acids were identified [67]. GABA, along with total flavonoids, total polyphenols, riboflavin, and *β*-glucan, was detected in germinated highland barley [73]. GABA and dietary fiber fractions were also analyzed in different varieties of germinated wheat (*Triticum aestivum*) [10].

#### 3.2.3. Bioactive Peptides

Germination significantly increases the content of peptides and free amino acids because of protein hydrolysis by endogenous enzymes [41,42,67], thereby contributing to an overall improvement in protein digestibility [4,21,22,64,86]. However, several studies have moved beyond mere quantitative increase to directly evaluate the biological activity of these compounds. For example, in *C. formosanum* sprouts, which were germinated and subsequently fermented, proteomic analysis was used to identify peptides that exhibited high free radical scavenging scores and were predicted as potential DPP-III/IV and ACE (angiotensin-converting enzyme) inhibitors, thus confirming their bioactive functionality [41]. Furthermore, GABA is a non-protein amino acid whose concentration drastically increases with germination [3,16,17,26,66,68,73,86], and has been consistently identified as a key bioactive compound with evaluated functions such as blood pressure reduction, relaxation induction, improved brain function, and strengthened immunity, as well as in vitro antioxidant capacity [26,75,86].

Research has systematically characterized bioactive nitrogenous compounds across germinated cereals, documenting free peptides in djulis, soluble proteins in wheat, and comprehensive protein content analysis in corn sprouts and roots (*Zea mays* L., hybrid FH-1036) [6,37,41,79]. Free amino acid profiling studies have established specific compositional patterns in germinated wheat and brown rice grains [31]. These investigations collectively establish distinct profiles of bioactive nitrogenous compounds during germination processes.

#### 3.2.4. Melatonin and Indolic Compounds

Melatonin (N-acetyl-5-methoxytryptamine) and other indolic compounds represent a less studied group that is of considerable biological importance in germinated grains. A detailed study of bioactive compounds in germinated amaranth (*Amaranthus cruentus*) was conducted, identifying tryptophan and indolic derivatives such as caffeoylquinic acid, *p*-coumaroylquinic acid, and feruloylquinic acid [80].

This group of compounds has received less attention in scientific literature on germinated grains, but existing data suggest their significant contribution to bioactive properties, particularly regarding their antioxidant capacity and neuroprotective potential.

#### 3.2.5. Vitamins and Bioavailable Minerals

Germination significantly enhances mineral bioavailability and vitamin content in cereal matrices, as evidenced by improved iron and zinc accessibility in germinated corn (Zea mays variety ZM607-MUTUTU-18A) and elevated vitamin C levels in germinated wheat [4,37].

Germinated grain vitamin profiles include various vitamins. Research detected 5-methyltetrahydrofolate (5-MTHF), *β*-carotene, lutein, vitamin C, and vitamin B2 in various germinated quinoa varieties (*Chenopodium quinoa* Willd.) [20]. Studies identified carotenoids, including lutein, zeaxanthin, *α*-cryptoxanthin, *β*-cryptoxanthin, *α*-carotene, and *β*-carotene in germinated yellow corn (*Zea mays* L., cultivar Suyu 29) [26,69]. Research also identified vitamin E (tocopherols and tocotrienols) and *γ*-oryzanols in germinated rice, along with anthocyanins and phytosterols [90,91].

#### 3.2.6. Antioxidant Enzymes

Germination induces activation of endogenous antioxidant enzymes such as superoxide dismutase (SOD), peroxidase (POD), catalase (CAT), and ascorbate peroxidase (APX) [6,15,74,75]. These proteins are susceptible to denaturation and inactivation by heat treatment (such as cooking or drying at high temperatures) to which sprouted grains are often subjected before consumption [44,53,81,92]. Therefore, their direct activity in the human body after ingestion is minimal.

However, their role during the germination process is essential for catalyzing a series of beneficial transformations. These enzymes contribute to the de novo synthesis of various bioactive compounds and the release of bound forms of others, such as polyphenols, flavonoids (e.g., rutin, quercetin, kaempferol), and avenanthramides [1,11,16,23,25,35,41,53,65,74,92,93]. For example, germination increases the production of phenylpropanoid pathway enzymes, which are crucial for the formation of flavonoids [74]. Likewise, the enzymatic activity of germination contributes to the drastic reduction of antinutrients such as phytic acid and tannins, which in turn improves the bioavailability of essential minerals [3,4,16,21,26,42]. The antioxidant impact on the human body derives mainly from the higher concentration and bioaccessibility of these thermostable (heat-resistant) bioactive compounds resulting from the germination process, rather than from post-ingestion enzymatic activity [49].

Research has documented comprehensive antioxidant enzyme profiles in germinated wheat, with studies identifying superoxide dismutase, catalase, ascorbate peroxidase, guaiacol peroxidase, and pyrogallol peroxidase in cultivar Belija [94]. Similarly, research detected peroxidase, superoxide dismutase, and catalase in variety Dilkash 2020 [83]. Germinated barley studies demonstrated similar enzymatic profiles, with variety CDC Copeland showing *α*-amylase, *β*-amylase, and *β*-glucanase [45], while variety Bojos exhibited *α*-amylase and *β*-glucanase activity [44]. Comparable enzyme profiles have been documented in other germinated cereals, including corn, rice, and oats [89].

Phytase activity during germination represents a key enzymatic mechanism for improving mineral bioavailability [2,4,8,21,22,31,38,50], as phytic acid (IP6), a predominant antinutrient in cereals, forms insoluble chelating complexes with divalent cations such as iron, zinc, calcium, and magnesium, drastically limiting their absorption in the gastrointestinal tract [4,8,21,21,38]. During germination, there is significant activation of endogenous phytases [2,8,21,22,23,50,95], enzymes that catalyze the hydrolysis of phytic acid to myo-inositol and inorganic phosphorus, and to less phosphorylated forms of inositol. This enzymatic degradation results in a substantial reduction in phytate content and, consequently, a notable improvement in the bioaccessibility and bioavailability of essential minerals, including calcium, iron, zinc, magnesium, potassium, copper, and manganese [1,4,23,31,50]. The decrease in phytic acid–mineral molar ratios (e.g., Phy:Zn below 15 and Phy:Fe below 1) is a key indicator of optimized mineral absorption post-germination [2,4,24,38]. Therefore, phytase action increases grain nutritional value and facilitates vital micronutrient assimilation.

#### 3.2.7. Diverse Phytochemicals

Besides the main bioactive compound groups, germinated grains contain various phytochemicals with important biological properties, particularly photosynthetic pigments, which have been extensively studied. Research has detected total chlorophyll, chlorophyll *a*, chlorophyll *b*, and carotenoids in germinated wheat, barley, and oats (*Avena sativa* L.) [94]. Also, studies identified these same pigments in germinated wheat (*Triticum aestivum* L., variety Dilkash 2020) [83].

Identified carotenoids include specific compounds such as lutein, zeaxanthin, *α*-cryptoxanthin, *β*-cryptoxanthin, *α*-carotene, and *β*-carotene in germinated yellow corn (*Zea mays* L., cultivar Suyu 29) [70]. Likewise, studies identified carotenoids along with chlorophyll *a* and chlorophyll *b* in germinated wheat [37].

Other phytochemicals of interest include saponins, phytosterols, and alkaloids, with phytosterols (*β*-sitosterol) and triterpenoids (24-methylenecycloartanol) identified in germinated rice [90], and condensed tannins, hydrolyzed tannins, and saponins characterized in germinated grains [2]. Additionally, research has systematically analyzed photosynthetic pigments and secondary metabolites in various germinated grains, including total chlorophyll, carotenoids, anthocyanins, phenolic content, and proline accumulation [36,96,97].

#### 3.2.8. Dietary Fiber

Germination activates a broad spectrum of endogenous hydrolytic enzymes [2,11,42,52,64,68,72,92], including *β*-glucanase [5] and fibrinolytic enzymes, which catalyze the degradation of complex macromolecules such as starch, proteins, and, crucially, cell wall components. This enzymatic action leads to a structural modification of dietary fiber, releasing previously bound compounds and, in certain cases, improving the solubility of fiber fractions [21,53,92]. Although the direct conversion of insoluble fiber to soluble fiber is not a universally strict process in all grains, germination significantly improves the balance between these fractions by degrading and altering non-starch polysaccharides [64] and *β*-glucans [5,52,53], making the components more bio accessible [42,64].

Technologically, this modulation is of paramount importance for bread making [10,21,44], especially in flours that form gluten networks [10,98]. Insoluble fibers, having a high water absorption capacity, can compete for the water necessary for gluten hydration [10,21], adversely affecting the formation of its network, which translates into a reduction in the specific volume [10,21,98] of the product and a less desirable crumb texture [10,98]. By promoting the hydrolysis and structural alteration of these fibrous components, germination attenuates this deleterious effect [10,44], facilitating better dough functionality and, therefore, optimizing the textural and volume characteristics of baked goods [2,21,99]. This enzymatic activation also contributes to an overall improvement in digestibility and other techno-functional properties of germinated grains [1,21,34,74,86].

Dietary fiber in germinated grains includes various non-digestible polysaccharides with significant functional properties and health benefits. Research quantified *β*-glucan concentrations in several germinated cereals, noting decreases during germination due to enzymatic degradation. In germinated hulled oats variety Barra, *β*-glucan content decreased 46.8% after 216 h, while in dehulled oats variety Meeri, concentrations decreased 55.9% after 156 h [85]. Similarly, in germinated highland barley, *β*-glucan content showed a reduction of 9.68% after 48 h of germination [73]. Despite these reductions, the remaining *β*-glucan concentrations are physiologically significant for cholesterol reduction and glycemic control, as dietary intakes of 3–4 g/day have been associated with measurable cardiovascular benefits.

Arabinoxylans represent another crucial component of dietary fiber in cereals, with germination showing positive effects on their accumulation [27]. In germinated wheat, arabinoxylan content increased by 33% after 120 h of germination. This increase is nutritionally beneficial as arabinoxylans contribute to intestinal health through their prebiotic properties and their ability to modulate glucose absorption. These compounds were comprehensively studied in seven germinated grain species: wheat, oats, barley, rye, sorghum, brown rice, and buckwheat, along with inositol phosphates (InsP4, InsP5, InsP6) which exhibit additional mineral bioavailability enhancement properties [21,27,38]. In addition, InsP6 and its less phosphorylated forms (InsP1-5) exhibit antioxidant functions due to their ability to chelate divalent cations [27].

Comprehensive dietary fiber fraction analysis reveals compound complexity in germinated grains, with research characterizing soluble dietary fiber, insoluble dietary fiber, and total dietary fiber in three germinated wheat varieties: hard red spring wheat, hard white wheat, and soft white wheat [10]. These fiber fractions’ functional significance lies in distinct physiological effects: soluble fiber contributes to cholesterol reduction and glucose metabolism regulation through increased intestinal viscosity [27,53], while insoluble fiber promotes intestinal transit and serves as a substrate for beneficial microbiota fermentation, producing short-chain fatty acids with anti-inflammatory and metabolic regulatory properties [48,100].

## 4. Factors Influencing the Accumulation of Bioactive Compounds

The germination process significantly affects bioactive compound synthesis and accumulation in seeds. Several factors influence this dynamic biochemical transformation, including temperature, moisture availability, light exposure, oxygen levels, and germination duration. These conditions modulate enzymatic activity and metabolic pathways, stimulating phenolic compound, vitamin, and other antioxidant production. Optimizing these parameters can enhance germinated seed nutritional and functional properties, making germination a valuable tool for improving food quality.

### 4.1. Genetic Factors

Cereal genotype significantly influences bioactive compound production [101], as phenolic compound amounts in seed samples are strongly influenced by genotype (variety/cultivar), implying that different varieties of the same cereal can produce variable amounts of these bioactive compounds [13]. Research found that barley cultivars differed in determined phytochemical content, antioxidant potential, and cholinesterase inhibitory activity, suggesting a genetic basis for these variations [102]. Studies used eight different cultivars, including spring and winter varieties, observing differences in phenolic acid and flavonoid composition.

Growing evidence indicates that stress stimuli, both biotic and abiotic (e.g., salinity [87,89], UV-B radiation [20,26,49,69,73,87], low temperatures [26,36,94], and electrical pulses [44,45,96]) consistently induce the accumulation of bioactive compounds such as polyphenols, flavonoids, CAROTS, and GABA, as well as the activation of antioxidant enzyme systems, regardless of the intrinsic tolerance of the crop. In inherently stress-tolerant species, such as quinoa, recognized as a halophyte, exposure to high concentrations of NaCl has been shown to increase polyphenol production and antioxidant enzyme activity in a dose-dependent manner, even acting as a “chemical eustress” that improves the profile of health-promoting compounds [89]. Similarly, in cereals such as highland barley (*Hordeum vulgare* L. var. *nudum* Hook. f.), UV-B radiation, an environmental stressor, stimulates the synthesis of flavonoids, polyphenols, and antioxidant enzymes as a protective response, even in sprouts [73].

For less tolerant crops, strategies such as priming or elicitation with phytohormones (e.g., gibberellic acid (GA_3_) [39,79,83,94], indoleacetic acid (IAA) [39,94], and salicylic acid (SA) [39,94]) or biostimulants (e.g., biochar [79,83]) induce moderate stress that activates the plant’s defense systems, improving its resilience and promoting the synthesis of protective secondary metabolites [36,39,83,94]. The magnitude of this accumulation is, however, highly variable and specific to the genotype, the type of stressor, and the intensity and duration of the stimulus, underscoring the need to optimize conditions to maximize the nutritional and functional value of the grains [18,35,36,53,73,89,94,103]. This phenomenon highlights that stress modulation, at appropriate levels, is a key tool for improving the bioactive profile in a wide range of crops.

### 4.2. Environmental Conditions During Growth

Phenolic compound levels in seeds are strongly affected by environmental conditions during growth. Soil composition, climate, and harvest maturity influence metabolic pathways involved in biosynthesis. These factors impact both the accumulation and profile of bioactive compounds, making environmental management crucial for enhancing the nutritional and functional quality of seed-derived products [13].

### 4.3. Germination Process Parameters

Germination is a key factor that can significantly increase the content of bioactive compounds [16], with specific parameters including germination temperature, which affects the activity of enzymes involved in the biosynthesis and release of bioactive compounds [36,47]. Moderate temperatures may be more effective for certain compounds [36]. Humidity: adequate relative humidity is essential for enzymatic activation and metabolism during germination, which indirectly influences the accumulation of bioactives [32,47]. Lighting: the duration and intensity of light, including UV-B light, can stimulate the production of compounds such as phenols and flavonoids in sprouts [36,47]. Optimal intensity is crucial [73]. Germination time: the content of bioactive compounds varies significantly with the duration of germination. There is an optimal time to maximize the accumulation of specific compounds such as polyphenols and GABA [16,47]. The pH of the soaking and germination medium can influence enzymatic activity and the solubility of bioactive compounds [38].

### 4.4. Other Processing Treatments

In addition to germination, other processes can affect the accumulation or release of bioactive compounds, with fermentation particularly enhancing the solubility and extractability of flavonoids and other phenolic compounds through microbial enzymatic activity [24,77]. Soaking can reduce some antinutritional compounds, but release water-soluble bioactive compounds [77]. Cooking (autoclaving, baking, steaming) can have variable effects, from degradation to the release of certain bioactive compounds, depending on temperature and time [38,65]. Irradiation (microwave, controlled UV-B radiation, plasma) can stimulate the synthesis of bioactive compounds or improve their extraction by altering cellular structures [47,70,104]. For example, UV-B irradiation can increase the content of flavonoids and polyphenols [65]. Plasma treatment can also influence growth and accumulation of phytochemicals [70].

### 4.5. Abiotic Stress

Exposure to mild to moderate stress conditions, such as salinity or suboptimal temperature, can activate defense mechanisms in the plant that result in greater production of secondary metabolites with antioxidant activity [36,89]. Understanding these factors has allowed the development of strategies to maximize the synthesis of specific bioactive compounds, leading to the concept of “directed germination” or “inducer-assisted germination”.

## 5. Molecular Mechanisms of Inducers for Bioactive Compounds Enhancement

### 5.1. Physical Inducers: Molecular Mechanisms

#### 5.1.1. Plasma-Activated Water (PAW)

Plasma-activated water operates through reactive oxygen and nitrogen species (ROS/RNS) generation, which function as molecular signaling molecules modulating plant metabolism [75,79,88,103]. The technology produces long-lived species including ozone, hydrogen peroxide, nitrates (NO_2−_ and NO_3−_), and nitric oxide [37,70,96,105] that serve dual functions as nitrogen sources and hormonal regulators controlling seed dormancy termination [37,45,75,94]. The acidic environment (pH 3.87) enhances cellular destabilization, while nitric oxide acts as an endogenous dormancy regulator, promoting germination initiation and activating secondary metabolite biosynthetic pathways [45,70,96,103,106]. This controlled abiotic stress triggers adaptive responses resulting in enhanced accumulation of phenolic compounds, chlorophylls, CAROTS, and GABA as cellular protective mechanisms [17,25,30,37,75].

#### 5.1.2. Pulsed Electric Field (PEF)

Pulsed electric field technology induces reversible electroporation of cellular membranes, enhancing permeability and facilitating nutrient uptake during germination [6,44,107]. The treatment generates controlled oxidative stress through ROS production, activating defensive metabolic pathways and stimulating bioactive compound biosynthesis, such as phenolics [6,44]. At the molecular level, PEF disrupts dipole–dipole interactions in protein secondary and tertiary structures, resulting in enzyme activation and enhanced bioavailability of essential micronutrients, including calcium for metalloenzyme function [44]. The electroporation of embryonic cell walls modifies ion transport mechanisms and influences enzymatic activities of *α*-amylase and *β*-glucanase, requiring precise parameter optimization to maintain germination viability [43,44,96,107].

#### 5.1.3. Ultraviolet Radiation (UV)

UV functions as an environmental stress signal that activates protective metabolic responses and stimulates secondary metabolite synthesis [18,92,96]. UV-B radiation (280–315 nm) specifically upregulates phenylalanine ammonia-lyase (PAL), cinnamic acid 4-hydroxylase (C4H), and 4-coumarate-CoA ligase (4CL) in the phenylpropanoid pathway [26,69,74,80], significantly enhancing gene expression and enzymatic activity for flavonoid and phenolic biosynthesis [26,74]. UV-A radiation (315–400 nm) activates antioxidant defense systems, thermal energy dissipation mechanisms, and chlorophyll quenching processes while promoting phenolic compound accumulation as stress adaptation responses [49]. UV-C radiation (200–280 nm) induces photooxidative damage to DNA, proteins, lipids, and sterols, simultaneously activating protective enzymes including peroxidase (POD) and PAL as cellular defense mechanisms [28,80].

#### 5.1.4. Magnetic Fields (MFs)

Magnetic fields, as physical inducers, modulate the accumulation of bioactive compounds during germination through specific molecular mechanisms. Exposure to MFs can alter the conformation of enzymatic proteins, such as GABA aminotransferase (GABA-T), reducing their activity and promoting the hyperaccumulation of GABA by decreasing its catabolism [15]. They also increase cell membrane permeability, facilitating the absorption of exogenous compounds and altering electrical conductivity and root hair formation [15]. On a broader level, electromagnetic fields can induce changes in phytohormone balance and protein expression and activate secondary metabolism, affecting the biosynthesis of compounds such as quercetin and the mineral content (Fe, Zn) in harvested seeds [103].

#### 5.1.5. Ultrasound Treatment (US)

US operates through acoustic cavitation, generating mechanical forces and thermal effects that modify grain microstructure by creating micropores and activating enzymatic systems [11,17,31]. Cavitation effects enhance cellular membrane permeability [22,95], facilitate water and oxygen diffusion [11,108], and activate genes associated with energy metabolism and transcriptional regulation [66,108]. The technology functions as an abiotic stress elicitor, elevating intracellular Ca^2+^ concentrations and pH while specifically activating glutamate decarboxylase (GAD), the enzyme responsible for glutamate-to-GABA conversion during stress responses [10,31,108]. Ultrasonic cavitation stimulates secondary metabolite production, including GABA, antioxidants, and phenolic compounds through adaptive cellular mechanisms, while concurrently activating PAL for enhanced phenolic biosynthesis [8,22,66].

#### 5.1.6. Light and Photoperiod Regulation

Light intensity and photoperiod manipulation serve as environmental signals regulating metabolic pathways for secondary metabolite biosynthesis [36]. Controlled illumination stimulates flavonoid and phenolic compound synthesis through metabolic reprogramming that favors bioactive compound accumulation [36,47]. Pulsed light treatment enhances cellular membrane permeability, increases glutamic acid availability, elevates glutamate decarboxylase (GAD) activity, and reduces *γ*-aminobutyrate transaminase (GABA-T) activity, thereby promoting GABA accumulation while stimulating phenylalanine biosynthesis and carbohydrate metabolism [9]. Extended photoperiods combined with controlled temperature stress activate defense mechanisms that enhance secondary metabolite synthesis, including phenolics, flavonoids, and antioxidant compounds [36].

#### 5.1.7. High Hydrostatic Pressure (HHP)

High hydrostatic pressure modifies protein–starch structural interactions, increases cellular permeability, and facilitates mass transport processes that enhance bioactive compound extractability. The treatment enables phenolic compound release from cellular matrices through protein complex structural modifications, resulting in enhanced antioxidant capacity and mineral retention without thermal degradation [98].

#### 5.1.8. Microwave Irradiation

Microwave technology activates key germination-associated enzymes and regulates antioxidant enzyme gene expression through controlled electromagnetic energy application [71,104]. Microwave irradiation enhances flavonoid biosynthesis pathway enzymes, specifically increasing phenylalanine ammonia-lyase (PAL), chalcone isomerase (CHI), and flavonol synthase (FLS) activities, resulting in elevated flavonoid content and antioxidant capacity [104]. Ultra-high frequency microwave electromagnetic fields (UHF EMFs) induce ROS production, triggering protective responses including enhanced synthesis of phenolic acids (caffeic, ferulic, vanillic, gallic, coumaric, syringic, and SA) [106]. The technology activates catalase and superoxide dismutase while regulating antioxidant enzyme gene expression, promoting comprehensive metabolic enhancement [71].

#### 5.1.9. Cold Plasma Technology

Cold plasma treatments generate ROS/RNS that directly interact with seed surfaces, with atomic oxygen species functioning as primary contributors to bioactive enhancement [105,109]. Surface barrier discharge (SBD) plasma induces controlled oxidative stress through ROS production, stimulating defensive metabolic responses and enhancing germination parameters [46]. Atmospheric pressure cold plasma creates surface modifications through “surface etching”, facilitating water absorption while activating biosynthetic pathways for phenolic compounds, tocopherols, *γ*-oryzanol, anthocyanins, and phytosterols [105]. The technology operates through surface micropore creation, chemical modification by reactive oxygen and nitrogen species (RONS), and hydrophilic surface alterations that enhance nutrient availability and metabolic activation [88,110].

#### 5.1.10. Gamma Irradiation

Gamma irradiation induces macromolecule breakdown through free radical-mediated hydrolysis of chemical bonds. The treatment fragments large starch and protein molecules into smaller components, enhancing digestibility and bioactive compound accessibility. This inducer significantly increases total phenolic content and antioxidant activity through controlled molecular degradation and structural reorganization [111].

#### 5.1.11. Controlled Thermal Processing

Controlled thermal treatments, including tempering and roasting, demonstrate synergistic effects with germination for phenolic compounds and antioxidant enhancement. Thermal processing modifies molecular structures while preserving bioactive compounds, achieving optimal parameters that balance bioactive enhancement with nutritional quality maintenance. These treatments exhibit enhanced effectiveness when integrated with controlled germination processes [51].

### 5.2. Chemical Inducers: Molecular Pathways

#### 5.2.1. Phytohormones

Gibberellic acid (GA_3_) promotes *α*-amylase and hydrolytic enzyme mRNA synthesis in aleurone layers, accelerating endosperm starch degradation through transcriptional upregulation [40]. Phytohormones including indole acetic acid, SA, and gibberellic acid function as elicitors generating controlled oxidative stress, inducing phenylalanine ammonia-lyase (PAL) activity and stimulating phenolic compound synthesis through phenylpropanoid pathway activation as antioxidant defense mechanisms [39,50,112]. Jasmonic acid and chitosan regulate secondary metabolite biosynthesis gene expression, activating phenylpropanoid pathway enzymes and enhancing gallic acid, catechin, chlorogenic acid, and rutin accumulation [112].

#### 5.2.2. Stress-Inducing Compounds

Slightly acidic electrolyzed water (SAEW) functions as a controlled stress factor activating dormant enzymes during germination, particularly glutamate decarboxylase (GAD), thereby increasing GABA synthesis. This stress initiates signal transduction cascades that modify cellular metabolism and enhance phenolic compound, flavonoid, and amino acid accumulation through controlled oxidative stress responses [25].

Hydrogen-rich water (HRW) operates as a molecular signaling agent, activating antioxidant enzymes and upregulating Dreb1 gene expression associated with drought tolerance, while reducing oxidative stress and modulating H_2_O_2_ and nitric oxide levels [113].

#### 5.2.3. Mineral and Salt Stress

Salt stress induced by NaCl modifies germination environment ionic strength, stabilizing enzyme three-dimensional structures and enhancing enzymatic activity. This stimulates phenolic compound accumulation and starch structural modification through controlled stress responses [72,89]. NaCl induces oxidative stress that activates antioxidant systems and upregulates specific genes including ZmPSY and ZmCYP97C in carotenoid biosynthetic pathways, while CaCl_2_ functions as a secondary messenger maintaining membrane structural and functional integrity and regulating carotenoid synthesis gene expression [87]. Salt stress mechanisms trigger enhanced ROS production, stimulating phenolic compound synthesis as cellular protection against oxidative damage [89].

#### 5.2.4. Plant-Derived Elicitors

Sucrose induces defense responses while serving as an energy and carbon source, stimulating secondary metabolite biosynthesis through shikimic acid-phenylpropanoid pathway activation. Calcium chloride functions as a secondary messenger in cellular signaling cascades, regulating bioactive compound synthesis and metabolic pathways [33]. Pyridoxal phosphate (PLP) activates glutamate decarboxylase (GAD), increases glutamate substrate availability, and positively regulates gene expression (GAD, GS1/2, and GOGAT), functioning as an enzymatic cofactor in amino acid metabolism for enhanced GABA production [30]. Citric acid stimulates phenolic biosynthetic pathways by enhancing phenylalanine ammonia-lyase (PAL) activity, the rate-limiting enzyme in phenolic compound synthesis [114].

### 5.3. Biological Inducers: Enzymatic and Microbial Mechanisms

#### 5.3.1. Concurrent Fermentation

Fermentation processes activate endogenous enzymes that degrade polysaccharides and proteins while producing microbial enzymes (protease, *β*-glucosidase, and amylase) that hydrolyze cellular matrices and liberate bioactive compounds [41,42]. *Rhizopus oligosporus* fermentation specifically enhances protein degradation, carbohydrate hydrolysis, and cell wall decomposition, facilitating bioactive compound release [41]. The process involves glutamate decarboxylase (GAD) activation under hypoxic conditions, catalyzing glutamate-to-GABA conversion, alongside activation of amylases, *β*-glucanases, phytases, and proteases for macronutrient degradation and phenylalanine ammonia-lyase for phenolic biosynthesis [5,24].

#### 5.3.2. Microbial Extracts and Enzymatic Enhancement

Fermentation and germination processes result in coordinated enzymatic activation during germination and microbial enzyme production during fermentation, degrading protein–starch matrices and enhancing bioactive compound release while reducing antinutrients [42,50]. The mechanisms involve phytate bond hydrolysis, mineral chelate disruption, and enhanced bioavailability through coordinated enzymatic activities [2,42]. Endogenous phytase activation during germination and soaking, combined with microbial phytase production during fermentation, effectively hydrolyzes phytates and enhances mineral bioavailability through specific enzymatic pathways [4]. US combined with fermentation demonstrates synergistic effects in activating enzymatic systems and enhancing bioactive compound liberation [3].

**Figure 3:**
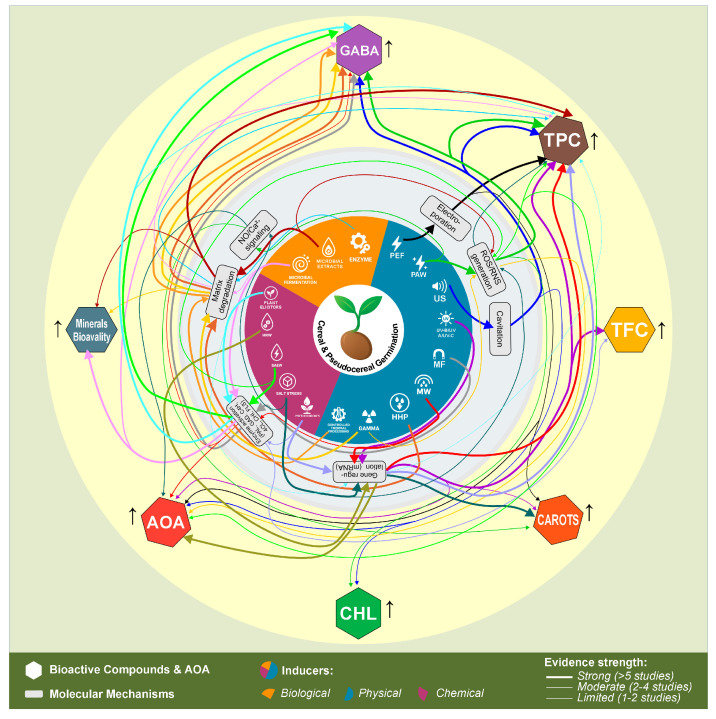
Comprehensive overview of inducer mechanisms for bioactive compound enhancement during cereal and pseudocereal germination. The diagram illustrates the molecular pathways through which physical inducers (blue section: PEF, pulsed electric field; US, ultrasound; UV, ultraviolet radiation; PAW, plasma-activated water; MF, magnetic field; MW, microwave; HHP, high hydrostatic pressure; Gamma, gamma irradiation; Thermal, controlled thermal processing), chemical inducers (purple section: phytohormones, salt stress, SAEW, slightly acidic electrolyzed water; HRW, hydrogen-rich water; elicitors), and biological inducers (orange section: microbial fermentation, microbial extracts, enzymes) activate key molecular mechanisms (gray boxes: electroporation, ROS/RNS generation, cavitation, gene regulation, enzyme activation, matrix degradation, NO/Ca^2+^ signaling) to enhance bioactive compounds (hexagons: GABA, *γ*-aminobutyric acid; TPC, total phenolic compounds; TFC, total flavonoid compounds; AOA, antioxidant activity; CAROT, carotenoids; CHL, chlorophylls; mineral bioavailability). Thick arrows indicate pathways supported by strong scientific evidence (>5 studies), while thin arrows represent pathways with moderate evidence (1–4 studies). All connections shown are based on experimental validation from 126 peer-reviewed studies (2015–2025). Arrow colors serve as visual guides to trace pathways from inducers to bioactive compounds and do not represent additional scientific classifications.

The mechanistic relationships between inducer categories and bioactive compound enhancement pathways are comprehensively presented in Figure 3. This integrated framework demonstrates how physical, chemical, and biological inducers converge through specific molecular mechanisms to enhance target bioactive compounds in germinated cereals and pseudocereals. The diagram illustrates the central role of enzyme activation, ROS/RNS generation, and gene regulation as key mechanistic bridges, with arrow thickness indicating the strength of supporting scientific evidence. This systematic overview provides the mechanistic foundation for rational inducer selection and optimization strategies in functional food development.

## 6. Emerging Physical Inducers

### 6.1. Controlled Germination

Temperature and germination time are critical parameters that significantly influence the germination of cereals and pseudocereals, as well as the enhancement of their bioactive compounds and antioxidant capacity [16,29,34,52,53,115,116,117] (Table 2). Optimal temperatures vary according to species: for example, finger and pearl millet show better growth at 30 °C, while buckwheat prefers lower temperatures (22 °C) [34] or 25 °C [36]. In quinoa, 20 °C for 42 h maximizes total phenolic content (TPC) with an 80% increase and antioxidant activity with a 30% increase [86]. However, elevated temperatures (60 °C) during drying can decrease TPC in red quinoa sprouts [29].

Germination time is also crucial; in quinoa, the most pronounced increases in bioactive metabolites occur between the third and fifth day [115], and GABA content doubles at 48 h [16]. Nevertheless, prolonged times can reduce phenols and antioxidant activity in some millets [34]. Initial soaking is essential to activate enzymes and solubilize antinutrients [16,22]; however, prolonged soaking in water can worsen some nutritional parameters in quinoa [16]. The light/dark regime also plays a role; an extended photoperiod (20 h light and 4 h dark) can stimulate pigment biosynthesis in buckwheat microgreens [36]. Germination in darkness was commonly used in studies [29,117].

Other parameters such as ultrasonication and microwave treatment, often combined with germination, can enhance the extraction of phytochemicals and antioxidant activity in millets [22,116]. The use of priming with GABA increased germination and antioxidant activity in aged wheat and triticale (*×Triticosecale* Wittm.) [75]. The general mechanism of action involves the activation of hydrolytic enzymes that release phenolic compounds and other bioactives, de novo synthesis of secondary metabolites, improvement in nutrient bioavailability, and activation of enzymatic and non-enzymatic antioxidant defense systems [14,16,22,29,52,53].

Systematic optimization of germination conditions constitutes a fundamental approach for enhancing bioactive compound profiles across diverse cereal and pseudocereal species (Table 3), while quinoa-specific protocols establish benchmarks for controlled germination methodologies (Table 2).

### 6.2. Plasma-Activated Water (PAW) Treatments

The application of plasma-activated water has emerged as an inducer with significant impacts on germination and enhancement of bioactive compounds, with studies revealing that PAW not only promotes germination in cereals such as wheat and barley but also enhances the accumulation of key bioactive compounds and antioxidant capacity [37,45]. Research demonstrated that optimal PAW treatment for 3 min (PAW-3) in wheat increased germination by up to 100%, with notable increases in chlorophyll *a* (89.46%), chlorophyll *b* (112.46%), carotenoids (91.58%), TPC (10.46%), and superoxide dismutase activity (47.12%), translating into robust antioxidant capacity (up to 35.34% by ORAC) [37]. Studies also observed improvements in germination and increased *β*-amylase activity in barley treated with PAW [45].

The underlying mechanism for bioactive compound enhancement is attributed to a synergistic combination of factors, including improved nitrogen supply from nitrates and nitrites present in PAW [37], induction of mild abiotic stress that activates metabolic defense pathways, and the signaling action of reactive oxygen and nitrogen species (RONS), which, at controlled concentrations, modulate gene expression and enzymatic activity involved in the biosynthesis of these valuable compounds [37,45]. These results establish PAW as a promising tool for producing functional foods by improving nutritional value.

### 6.3. High Hydrostatic Pressure (HHP) Treatments

High hydrostatic pressure treatments demonstrate variable effects on cereals depending on the specific technology and conditions employed, with traditional HHP combined with soaking significantly enhancing bioactive compound content, as evidenced by buckwheat flour showing a 16.1% increase in total phenolic content when treated with one cycle of HHP (600 MPa, 30 min) following soaking pretreatment [98]. The mechanism involves improved cell permeabilization and enhanced extractability of phenolic compounds through pressure-induced mass transport facilitation.

In contrast, high pressure carbon dioxide (HPCD) treatments showed predominantly negative effects on germination capacity. HPCD significantly reduced oat germination from 58% to 0% and completely inhibited barley germination under treatment conditions [120]. The mechanism involves dissolved CO_2_ penetrating seeds, modifying cellular pH, and forming bicarbonate complexes that affect key enzymes such as *α*-amylase, with water activity being a critical determining factor.

These findings indicate that while traditional HHP with soaking proves effective for enhancing bioactive compound extractability, CO_2_-based pressure treatments require careful optimization to avoid detrimental effects on seed viability, suggesting that pressure treatment outcomes are highly dependent on the specific methodology and target cereal species [98,120].

### 6.4. Pulsed Electric Fields (PEFs)

Regarding germination, PEF treatment can exert both stimulating and inhibitory effects, depending on the treatment parameters and pre-existing conditions of the seed. It was observed that the application of PEFs at 6 kV·cm^−1^ with 50 pulses increased wheat seed germination, an effect that is directly associated with increased water uptake induced by cell membrane permeabilization [6]. Similarly, it was reported that PEF treatments of lower intensity (3 kV·cm^−1^, 9.9–19.8 kJ/kg) applied before the first hydration cycle in wheat improved germination parameters [44]. However, it is noted that prolonged pre-soaking before PEF treatment can be detrimental to germinative energy in barley, suggesting an optimal window of PEF application to favor germination without compromising embryo viability [107].

Regarding the enhancement of bioactive compounds, research indicates a positive impact of PEFs on the accumulation of valuable metabolites. Significant increases in CAROTS were found in the juice of wheat seedlings treated with PEFs, a 34% increase with treatment at 6 kV·cm^−1^ and 50 pulses [6]. Significant increases in total phenolic compound content (18.56%) and chlorophylls (373%) were also reported.

Notable increases in *α*-amylase activity (up to 104%) and *β*-amylase (up to 25%) with PEF treatments (3 kV·cm^−1^, 9.9–19.8 kJ/kg) have been reported in wheat malting [44]. These findings highlight PEFs’ ability to modulate the production of key enzymes with industrial applications.

Antioxidant capacity, evaluated by the DPPH assay, was also significantly increased (5.78%) in the juice of wheat seedlings treated with PEFs, which correlates with the increase in phenolic compound content and other metabolites with antioxidant properties [6].

The underlying mechanism of action for these various effects of PEFs focuses on cell membrane permeabilization [6,44,107], where the application of high voltage, short-duration pulses induces an increase in transmembrane potential, leading to the formation of micropores in the cell membrane and facilitating mass transport and water absorption [44]. This process can be reversible or irreversible depending on the treatment intensity. Permeabilization can also trigger stress responses in the plant, including the production of reactive oxygen species (ROS), which in turn can activate defense mechanisms that lead to the accumulation of antioxidant compounds and other secondary metabolites, such as CAROTS, total phenolic compounds, and chlorophylls [6]. Additionally, PEF can directly influence enzyme activity and synthesis by altering protein structure or facilitating cofactor availability [6,44].

PEF technology represents a promising tool for modulating germination, enriching key bioactive compounds (including enzymes and antioxidants) in cereals and pseudocereals. Optimization of PEF parameters, considering factors such as electric field intensity, number and duration of pulses, as well as seed pretreatment conditions (e.g., hydration level), is crucial for directing PEF effects toward desired outcomes in various food industry applications.

### 6.5. High Voltage Electric Fields (HVEFs)

Treatment with high voltage electric fields has demonstrated potential for enhancing germination parameters in cereals, though research on bioactive compound enhancement remains limited. Pre-sowing stimulation using constant, alternating, and pulsed high voltage electric fields improved germination speed and uniformity in winter triticale and barley, with optimal constant high voltage electric field (CHVEF) treatment at 3 kV·cm^−1^ for 60s achieving 96.7% germination energy and 98.7% uniformity. While significant improvements in growth parameters were observed, including 28.7% increase in root system length and 31.0% increase in grains per spike in triticale, no specific bioactive compounds were analyzed in these studies [121].

The stimulating mechanism involves redistribution of electrical charges within the seed’s internal structure, altering physicochemical processes and intensifying biological activities. Although HVEFs showed greater resistance to drought stress, which could be indirectly related to oxidative stress mechanisms, antioxidant activity and specific bioactive compound accumulation were not quantified [121]. Further research is needed to evaluate the potential of HVEF treatments for enhancing bioactive compound content in germinated cereals, as current studies focus primarily on germination and growth performance rather than phytochemical enrichment.

### 6.6. Magnetic Fields

Studies reveal significant effects of magnetic fields on germination and the accumulation of bioactive compounds [15,122]. In triticale seeds, the application of magnetic fields in the range of 2.23–3.72 mT (millitesla) accelerated germination, evidenced by the decrease in time required to reach 50% germination and the achievement of final germination rates above 90% [122]. The magnetic time model explains this phenomenon. In contrast, in germinated brown rice treated with a 10 mT magnetic field in the presence of exogenous GABA, no significant promotion of root growth was observed [15].

A notable finding is the substantial increase in GABA content in magnetically treated GBR with exogenous GABA supplementation, reaching increases in levels from 56% to 207%. Regarding antioxidant capacity, magnetic treatment had a modest effect on the activity of antioxidant enzymes in GABA-enriched GBR, suggesting that the increase in GABA content is the dominant factor in modulating the antioxidant response [15]. The proposed mechanism of action for germination acceleration involves an interaction with temporal processes within the seed [122]. On the other hand, it has been elucidated that the increase in GABA is mainly due to an improvement in cell membrane permeability, facilitating the absorption of exogenous GABA, with a possible minor contribution from the inhibition of the GABA-aminotransferase enzyme [15].

Considering the evidence presented, magnetic fields demonstrate a positive effect on accelerating triticale germination and significant potential for increasing GABA content in germinated brown rice in the presence of exogenous GABA, primarily through improved cell membrane permeability.

### 6.7. High Pressure Carbon Dioxide (HPCD)

The findings presented suggest that HPCD demonstrates limited potential as a germination enhancer, showing predominantly inhibitory effects on seed viability across cereal types, with treatment conditions, especially hydration, exacerbating the negative impact on germination rates [120]. Further research is needed to optimize HPCD parameters that could potentially balance antimicrobial efficacy with preservation of germination capacity.

### 6.8. Microwave Irradiation

Microwave irradiation proved to be an effective strategy to enhance the accumulation of bioactive compounds in cereals and pseudocereals during germination, with tartary buckwheat showing a significant 31.78% increase in total flavonoid content in sprouts following microwave treatment (300 W/50 s) compared to the control [104]. This increase correlated with higher activity of key enzymes in flavonoid biosynthesis, such as phenylalanine ammonia-lyase (PAL), chalcone isomerase (CHI), and flavonol synthase (FLS) [104]. Similarly, exposure to microwaves (600 W/30 s) stimulated the total flavone content in tartary buckwheat (*Fagopyrum tataricum*) sprouts [71]. In barley seedlings, microwave treatment increased the total amount of phenolic substances with antioxidant properties [106].

The proposed mechanism of action involves the ability of microwaves to penetrate seed tissues, altering macromolecular structure [104,116] and affecting physicochemical characteristics [104]. Additionally, it is suggested that microwave irradiation induces the accumulation of stress-related transcription factors and the expression of key genes for flavonoid biosynthetic enzymes [100]. Finally, an increase in tyrosinase and acetylcholinesterase inhibitory activities was observed, which could also be related to the higher concentration of phenolic compounds and flavonoids [71,104].

Controlled microwave irradiation application during germination emerges as a promising technique to enrich cereals and pseudocereals with key bioactive compounds, mainly flavonoids and other phenols, through biosynthetic enzymatic pathway modulation, suggesting added value for functional food production.

### 6.9. Light Intensity

Controlled visible light modulation during germination represents a precise biotechnological strategy for enhancing bioactive compound profiles in cereals and pseudocereals. This approach demonstrates significant potential for developing superior functional foods through targeted secondary metabolite accumulation.

In common buckwheat (*Fagopyrum esculentum*), photoperiod manipulation emerges as a critical factor. Extended photoperiod conditions (20/4 h light/dark) increased total chlorophyll content by 35.40% and total CAROTS by 21.34%, while maximizing total flavonoid production and antioxidant activity [36]. Similarly, tartary buckwheat germination under optimized light intensities (6000–10,000 lux) enhanced rutin, flavonoids, and total polyphenol accumulation, with maximum antioxidant capacity achieved at 10,000 lux [47]. Blue corn further demonstrates light’s critical role, with germination under controlled light/dark cycles using white fluorescent tubes (16 W/2700 K) resulting in a 9.9% increase in total anthocyanin content, confirming the importance of visible light in pigment biosynthesis during seedling development [26,92].

These findings establish that precise control of light intensity and duration constitutes a powerful tool for optimizing the functional and nutritional quality of cereal and pseudocereal sprouts through quantifiable bioactive compound accumulation.

### 6.10. Pulsed Light (PL)

Pulsed light (PL) emerges as an effective technology to positively influence cereal germination, as demonstrated by studies on germinated brown rice and corn [9,68]. In brown rice, pulsed light treatment (PLT) significantly increased sprout length between 12.7% and 26.9% in eight varieties, while in corn, PL promoted germination and accelerated macromolecule hydrolysis [9,68].

Regarding the enhancement of bioactive compounds, GABA stands out as the main enriched compound, with germinated brown rice showing increases over 100% in the eight varieties analyzed (being most significant in the Koshihikari variety) [68], while germinated corn demonstrated a 27.20% increase in GABA content after pulsed light treatment [9].

The pulsed light mechanism varies according to the cereal. In brown rice, PLT is proposed to activate metabolic pathways related to phenylalanine biosynthesis, carbohydrate and energy metabolism, as well as the GABA shunt pathway and polyamine degradation, with the OsbZIP56 transcription factor playing a key regulatory role [68]. In germinated corn, the mechanism involves the activation of the glutamate decarboxylase (GAD) enzyme, crucial for GABA synthesis from glutamic acid, and the inhibition of *γ*-aminobutyric transaminase (GABA-T), which degrades GABA, leading to its accumulation. Metabolomic analysis in corn revealed the activation of metabolic pathways associated with amino acid and carbohydrate metabolism, which influence GABA production [9].

Considering the evidence presented, pulsed light is an effective tool for promoting germination and significantly enriching GABA content in cereals such as brown rice and corn, by modulating specific metabolic pathways and activity. The electromagnetic and pressure technologies discussed in the preceding sections, including their optimal parameters, mechanisms of action, and quantitative results for bioactive compound enhancement, are comprehensively detailed in Table 4.

### 6.11. Ultraviolet Radiation (UV)

UV radiation application can influence the germination process of cereals and pseudocereals, enhancing the accumulation of bioactive compounds and antioxidant capacity [28]. In some cases, UV-C radiation did not affect germination yield and even decreased the time needed to reach commercial height in chia (*Salvia hispanica* L.) [28].

Bioactive compound enhancement demonstrates significant results across multiple species, with blue corn showing substantial increases when germination was combined with UV-B elicitation: TPC increased by 587.2%, total anthocyanins by 29.9%, and GABA by 199.9% [26]. Amaranth sprouts treated with UV-C showed a 17.7% increase in *p*-coumaroylquinic acid [80]. In buckwheat, optimized UV-B treatment increased total flavonoid content by 97% [74]. Germinated highland barley exhibited polyphenol level increases up to 49.40% under specific UV-B radiation conditions [73].

Antioxidant capacity improvements were observed across multiple assays, with blue corn germinated and elicited with UV-B showing antioxidant activity increases of 133.9% by ABTS and 173.4% by DPPH [26]. UV-C radiation positively influenced chia sprout antioxidant properties, with significant increases in DPPH activity [28]. UV-B treatment also improved buckwheat antioxidant capacity [74].

UV radiation induces stress in seeds and seedlings, activating metabolic defense pathways including the phenylpropanoid pathway, crucial for synthesizing phenolic compounds and flavonoids that protect against UV damage [73,74]. UV radiation generates reactive oxygen species (ROS), which stimulate the activity and expression of antioxidant enzymes such as superoxide dismutase (SOD), peroxidase (POD), catalase (CAT), and ascorbate peroxidase (APX), strengthening the plant’s antioxidant defense system [74].

UV radiation applied in a controlled manner during germination emerges as an effective strategy to enrich germinated cereals and pseudocereals with key bioactive compounds and enhance their antioxidant capacity. However, optimizing exposure conditions is crucial to avoid negative effects on germination and growth.

### 6.12. Cold Atmospheric Plasma

Cold Atmospheric Plasma (CAP) treatment emerges as a technology with diverse effects on the germination of cereals and pseudocereals, with dielectric barrier discharge (DBD) plasma demonstrating improved rice germination through a maximum 9.0% increase in germination rate at 60 s exposure, in addition to increasing vigor index and germination speed [123]. Similarly, it was found that an optimal 6 min plasma exposure increased the wet basis of barley seedlings by 137.5% compared to the control [70].

Variable field performance presents challenges for practical applications, as although in vitro buckwheat germination was unaffected, seedling emergence in field conditions decreased between 11% and 20%, suggesting that laboratory effects do not always translate to field applications [103]. Short treatments (8.7 s) with Ar-O_2_ and Ar-air plasma post-discharge improved root systems of wheat, barley, and rye, increasing root mass by up to 16.2% in barley [105]. Conversely, Cold Atmospheric Pressure Plasma (CAPP) can harm barley germination with increasing doses and prolonged exposure times, completely inhibiting germination with nitrogen plasma at 60 s or more [88].

Bioactive compound enhancement shows promising results, with single 6-min plasma exposure significantly increasing soluble sugars, free amino acids, and key secondary metabolites in barley sprouts, including saponarin (50%), GABA (90%), and policosanols (90%) [70]. Biochemical composition alterations in harvested buckwheat included changes in Fe, Zn, and quercetin content [103]. Cold plasma accelerated the time to reach maximum *γ*-oryzanol content in germinated rice and increased total vitamin E content in certain cultivars [90].

Antioxidant capacity results varied by species and treatment, with no significant differences found between plasma-treated and untreated germinated rice, although activity was higher in both groups compared to brown rice [90]. Cultivar-dependent changes in free radical scavenging activity in buckwheat treated with plasma and electromagnetic field were evidenced [103].

The mechanism of action involves seed surface modification, as observed by scanning electron microscopy (SEM) in rice [90,105,123], with this modification increasing hydrophilicity and water absorption, which in turn accelerates germination [48,123]. Plasma stimulates enzyme activity related to germination [88] and secondary metabolism [70]. Reactive oxygen and nitrogen species (RONS) generation plays a crucial role, acting as signaling molecules that modulate germination pathways and oxidative stress, though excessive exposure can be detrimental [88].

Cold plasma has the potential to positively influence germination and bioactive compound content in cereals and pseudocereals through seed surface modification and metabolic process activation, although effects vary significantly according to species, cultivar, and treatment parameters.

### 6.13. Ultrasonication

Ultrasonication has been investigated as a technique to stimulate the germination of cereals and pseudocereals while enhancing the accumulation of bioactive compounds and antioxidant capacity. With germination, enhancement demonstrates broad applicability. Ultrasonication accelerates the process and increases germination rates in wheat, brown rice, corn, and oats [17,31,108].

Bioactive compounds show consistent GABA enhancement across species, with significant GABA content increases observed in ultrasonically treated germinated cereals, including wheat (up to 30.7% increase in buckwheat) [10,11], red rice (*Oryza sativa* L.) [11,31], brown rice [17,108], and corn (30.55% more) [17]. In germinated oats, ultrasonication also enhanced the accumulation of avenanthramides [11]. Additionally, ultrasound treatment can increase the total phenolic compound content in germinated oats (11.24% more at 24 h) and brown rice [11,31], as well as proline in brown rice [31].

Antioxidant capacity improvements were measured using multiple assays. Ultrasonication improved DPPH free radical scavenging activity in germinated oats and brown rice (72.45% increase at 24 h in brown rice) [11,31]. Ferric reducing antioxidant power (FRAP) in brown rice showed non-significant increases [31].

The mechanism of action involves multiple cellular modifications. Ultrasonic waves induce mechanical stress and cavitation effects, altering cell membrane permeability and facilitating water entry. The treatment increases activity of endogenous enzymes such as glutamic acid decarboxylase (GAD) [10,11,17,31], stimulates metabolic pathways such as the GABA-shunt pathway, and activates antioxidant defense mechanisms in seeds [11,17]. Ultrasonication affects grain microstructure, increasing substrate availability for enzymatic hydrolysis [17,31].

Ultrasonication emerges as a promising strategy to improve germination and the nutritional value of cereals and pseudocereals by increasing key bioactive compounds and antioxidant capacity through multifactorial mechanisms related to physical stress and metabolic activation.

The radiation, plasma, and ultrasound technologies discussed above, including their optimal parameters, mechanisms of action, and quantitative effects on bioactive compound enhancement, are comprehensively summarized in Table 5.

## 7. Chemical Inducers of Germination

### 7.1. Plant-Derived Inducers

Plant-derived elicitors demonstrate remarkable efficacy in modulating secondary metabolite biosynthesis during cereal and pseudocereal germination [39,91,112]. Chitosan treatment (0.1%) induced a 23% enhancement in total phenolic content in buckwheat, while jasmonic acid (150 *μ*M) elicited a more pronounced 148% increase in phenol accumulation [112]. The enhanced metabolite profile consisted of gallic acid, rutin, catechin, chlorogenic acid, and (-)-epicatechin, indicating a coordinated upregulation of phenylpropanoid pathway enzymes. Both elicitors activate secondary metabolite accumulation through defense response induction and stimulation of biosynthetic enzymes, whereas salicylic acid demonstrates no measurable effect on phenolic biosynthesis [112].

Vegetable ashes (immature banana peel ash) employed as a plant-derived mineral source in corn malting significantly enhanced antioxidant properties and phenolic/flavonoid accumulation in the Coca-sr variety through enzymatic cofactor provision, antinutrient–protein complex disruption, and phytase activation. This multi-target mechanism facilitates metabolic optimization by eliminating inhibitory factors while simultaneously enhancing enzymatic efficiency [2].

These findings establish plant-derived inducers as potent biotechnological tools for targeted enhancement of phenolic compounds in germinated grains, operating through distinct yet complementary molecular mechanisms that optimize secondary metabolite accumulation and antioxidant capacity.

### 7.2. Minerals and Trace Elements

The application of minerals and trace elements during the germination of cereals and pseudocereals exerts significant effects on the accumulation of bioactive compounds and antioxidant capacity. Salt stress induced by NaCl in yellow corn (*Zea mays*) and quinoa has proven to be a key factor in enhancing secondary metabolites with antioxidant activity [87,89].

In yellow corn, although it negatively affected germination and growth, treatment with NaCl (300 mM) increased antioxidant capacity measured by DPPH and ORAC [87]. Similarly, in quinoa, salt stress (300 mM NaCl) induced a substantial increase in total polyphenol content (approximately 152%) and antioxidant activity, with a notable increase of 3700% in DPPH radical scavenging activity [89]. This increase is attributed to a defense mechanism where the plant increases the production of phenolic compounds to counteract the oxidative stress generated by salinity [89]. In wheat, NaCl stress during germination also resulted in a significant increase in total phenolic content (up to 243% at 48 h), associated with higher antioxidant activity, suggesting that salt stress can stimulate metabolic pathways leading to the synthesis of these compounds [72].

Supplementation with other minerals also influences biochemical composition. In yellow corn, the addition of CaCl_2_ (5 mM), besides mitigating the negative effects of NaCl on germination, also increased lutein content (up to 37%) and improved antioxidant capacity, through the regulation of genes involved in carotenoid biosynthesis [18]. Similarly, in buckwheat sprouts, the application of sodium silicate (SIL) and iron chelate (SIL-Fe) was shown to modulate the phenolic compound profile, with an increase in certain flavonoids (iso-rhamnetin, vitexin) and phenolic acids (ferulic, chlorogenic, sinapic). This effect is explained by elicitation, a method that induces the accumulation of secondary metabolites in plants [91].

Mineral and trace element availability manipulation during germination emerges as an effective strategy to modulate bioactive compound content and antioxidant capacity in cereals and pseudocereals, through activation of stress response mechanisms and specific metabolic pathways.

### 7.3. Plant Growth Regulators

Plant growth regulators, phytohormones, and vitamin B6 (pyridoxal phosphate) significantly modulate germination and bioactive properties in cereals and pseudocereals. Gibberellic acid (GA_3_) promotes germination in wheat by accelerating *α*-amylase activity and endosperm degradation, while abscisic acid (ABA) exerts inhibitory effects [40]. The exogenous application of IAA, SA, and GA_3_ at low concentrations stimulates growth and enhances antioxidant capacity in wheat sprouts. The combination of IAA (0.01 mg/mL), GA_3_ (0.001 mg/mL), and SA (0.001 mg/mL) synergistically increases antioxidant activity (FRAP 108%, DPPH 106%) and phenolic compounds (128% total phenols, 182% flavonoids) by activating enzymatic and non-enzymatic antioxidant defenses [39].

Pyridoxal phosphate (PLP) plays a crucial role as a cofactor for glutamate decarboxylase (GAD) in the synthesis of GABA. In germinated buckwheat, PLP treatment increases GABA content up to 867% compared to non-germinated, significantly enhancing antioxidant capacity (DPPH +15.52%, ABTS +31.47%) and antihypertensive capacity through angiotensin-converting enzyme inhibition, as well as increasing polyphenol content (+10.72%) [30].

These regulators constitute effective tools for modulating germination and enhancing bioactive compounds in cereals and pseudocereals through regulation of key enzymes and specific metabolic pathways.

### 7.4. Synthetic Chemical Inducers

Synthetic chemical inducers, for the purposes of this review, comprise compounds or solutions prepared in the laboratory, not directly derived from biological sources, that are applied during germination to stimulate specific metabolic responses in cereals and pseudocereals [25,33,38,113]. These agents, such as hydrogen-rich water [113], sucrose solutions with CaCl_2_ [33], acidic media [25], and electrolyzed water [25,76], cause moderate oxidative stress that activates secondary biochemical pathways, resulting in greater accumulation of bioactive compounds and improved antioxidant capacity [25,33,89,113].

Hydrogen-rich water (HRW) favored the accumulation of chlorophyll and soluble protein, crucial for growth and stress tolerance. The mechanism of action could be related to hydrogen’s ability to modulate oxidative stress in plants [113].

In buckwheat sprouts, the combined application of sucrose (3%) and CaCl_2_ (7.5 mM) significantly increases the content of polyphenols and total flavonoids. This treatment notably improves DPPH and ABTS radical scavenging, reducing power, and inhibition of lipid peroxidation by activating key enzymes in the phenylpropanoid pathway, such as phenylalanine ammonia-lyase (PAL) and tyrosine ammonia-lyase (TAL) [33].

Acidic medium use during germination has also been shown to enhance bioactive compounds. Research reported that brown rice germination under acidic conditions (pH 2.0–2.7) reduces phytic acid content and increases phytase activity, enhancing the bioaccessibility of minerals such as calcium, iron, and zinc [38]. Pretreatment with citric and lactic acid in adlay (*Coix lacryma-jobi* L.) germination has shown that citric acid was particularly effective in increasing total polyphenol content (TPC) by 119.6%, total flavonoid content (TFC) by 209.7%, and antioxidant capacity (ORAC) by 646.8% compared to raw adlay. The suggested mechanism is the stimulation of the phenolic biosynthetic pathway by citric acid [114].

Slightly acidic electrolyzed water (SAEW) is particularly effective in brown rice, significantly increasing antioxidant capacity (DPPH 839.7%, ABTS 792.2%, FRAP 934.2%), total phenols (746.1%), and total flavonoids (579.7%) compared to raw grain. This treatment also increases the levels of GABA, ferulic and *p*-coumaric acids, quercetin, and ascorbic acid, possibly through the activation of latent enzymes in response to mild oxidative stress [25].

Gaseous ozone (O_3_) has been investigated as an inducer in cereals, mainly for its fungicidal effect and its influence on germination [124]. The effect of different durations of O_3_ exposure (50 ppm, 1 L min^−1^, 1–5 hours) in wheat and other seeds has been evaluated [19]. The findings revealed that O_3_ treatment exerted a predominantly adverse impact on total phenolic compound content (TPC), with an average decrease of 39.4% in treated wheat grains compared to controls. A similar reduction was observed in wheat sprouts after ozone exposure. Regarding AOA, although germination generally increases AOA in sprouts compared to dry seeds, prolonged exposure to O_3_ (4–5 h) resulted in a significant decrease (*p* < 0.05) of AOA in wheat sprouts [19]. The proposed mechanism suggests that O_3_, due to its potent oxidative capacity, induces the production of reactive oxygen species (ROS) that could initially stimulate antioxidant synthesis; however, prolonged exposures or elevated concentrations cause over-oxidation, degrading phenolic compounds and other antioxidants [19,124]. The effects of O_3_ show clear dose-dependence: low concentrations preserve germination viability while high doses significantly reduce it [124]. It is crucial to more thoroughly investigate the optimal exposure conditions of O_3_ for cereals, determining specific parameters that avoid negative impacts on their biochemical profile and antioxidant capacity.

The findings presented suggest that synthetic chemical inducers constitute a promising strategy for significantly increasing bioactive compounds and antioxidant capacity in germinated cereals and pseudocereals, by modulating specific metabolic pathways and stress response. Optimization of concentration and exposure times is crucial to maximizing these benefits without compromising germination.

### 7.5. Nanomaterials

Nanomaterials represent an emerging technological approach for enhancing cereal and pseudocereal germination and bioactive compound accumulation. Zinc oxide nanoparticles (ZnO NPs) have demonstrated significant stimulating effects on germination of cereals such as pearl millet and corn. In pearl millet, seed priming with 150 ppm ZnO NPs improved germination by 20% and vigor index by 51% under laboratory conditions [125]. Similarly, in corn, ZnO NPs significantly improved germination, recording 92% compared to 68% for the control [97]. This effect is attributed to the nanoparticles’ ability to stimulate pre-germinative metabolism and improve tolerance to various abiotic stresses [125].

Regarding bioactive compound potentiation, an increase in chlorophyll content was observed in both species. In pearl millet treated with 150 ppm ZnO NPs, chlorophyll *a* and *b* levels increased by 12.13% and 11.22%, respectively, compared to the control [125]. Likewise, significant increases in chlorophyll content, sugar, proline, and phenolic compounds were reported in corn seedlings treated with ZnO NPs, suggesting improvement in various secondary metabolites [97]. Regarding antioxidant capacity, pearl millet extracts treated with ZnO NPs at 150 ppm exhibited DPPH radical inhibition of 78.11%, representing an 8.74% increase compared to the control (71.83%) [125].

Silver nanoparticles (AgNPs) synthesized from cyanobacterial extracts constitute another promising nanomaterial approach. These biologically synthesized AgNPs positively influenced germination of cereals such as barley (cvs. Giza-123, Giza-2000) and wheat (cvs. Benisweif-7, Misr-3), evidencing relative increases in germination percentages, germination rate index (GRI%), and germination velocity coefficient (GVC%), along with slight reductions in mean germination times (MGTs). Treatment with AgNPs based on cyanobacterial extracts showed superior promoting effects on germination attributes compared to extracts alone. While specific bioactive compound enhancement data for AgNPs were not detailed, their dual antimicrobial and germination-promoting properties suggest potential for developing multifunctional seed treatments [81].

The proposed mechanisms of action suggest that both ZnO and AgNPs, due to their small size and large surface area, enhance absorption of essential micronutrients for plant growth and metabolism, including chlorophyll synthesis and activation of antioxidant enzymes [97,125]. Additionally, they can influence gene expression related to germination and stress responses, while AgNPs may act as protective particles or carriers of bioactive substances [81,125]. These findings establish nanomaterials, particularly ZnO and biologically synthesized AgNPs, as promising strategies to improve germination and enhance key bioactive compounds in cereals, offering significant benefits for sustainable agriculture and improved nutritional quality of crops.

The chemical and biochemical inducers discussed above, including their optimal processing parameters, mechanisms of action, and quantitative effects on bioactive compound accumulation, are systematically summarized in Table 6.

## 8. Biological Inducers of Germination

The combination of germination and fermentation emerges as a synergistic biotechnological strategy to enrich the bioactive compound profile and enhance antioxidant capacity in various cereals and pseudocereals [5,41,42]. In naked barley, germination followed by fermentation significantly increased GABA content up to 10,30%, while maintaining high levels of *β*-glucan (5.66%) and improving antioxidant properties and total phenolic compound content (TPC) [5]. Similarly, in djulis sprouts, large-scale bioreactor fermentation (BF) preceded by four days of germination, demonstrated a notable increase in free peptide content and hydrolytic enzyme activity (amylase, glucosidase, and proteinase), suggesting the release of bioactive compounds and the generation of new metabolites. This process also elevated the levels of phenolic compounds, CAROTS, chlorophyll *a*, chlorophyll *b*, and anthocyanins [41].

In amaranth, although the study focused on optimization of germination, an increase in proteins, antioxidants, and dietary fiber was observed, along with a reduction in antinutritional factors such as phytic acid and tannins, and an improvement in fatty acids such as oleic and linoleic [50]. On the other hand, in red sorghum and pearl millet, the combination of germination and spontaneous fermentation resulted in a considerable reduction of phytates and an improvement in mineral status; however, antioxidant activity measured by DPPH was diminished [24]. Nevertheless, in contrast, in the case of foxtail millet, the combined application of germination and fermentation showed the most pronounced increase in antioxidant activity evaluated through different assays: DPPH (81.54%), FRAP (33.46%), and reducing power (184.52%) expressed as mg of ascorbic acid equivalent (AAE) per 100 g of dry flour [42].

The fundamental mechanism of action underlying these beneficial effects lies in the activation of endogenous enzymes during germination and the production of microbial enzymes during the fermentation process [5,41,42]. These enzymes catalyze the hydrolysis of complex macromolecules, releasing bioactive compounds that were previously bound or inaccessible, as is the case with the increase in GABA through the activation of glutamate decarboxylase (GAD) [5]. Fermentation also contributes to the degradation of antinutritional factors such as phytates and tannins, which indirectly can improve the bioavailability of other nutrients and release endogenous enzymes that participate in the modification of cellular components and the generation of compounds with greater antioxidant activity [42].

The strategic combination of germination and fermentation represents an effective methodology to optimize bioactive profiles and antioxidant capacity of cereals and pseudocereals through enzymatic activation and metabolic modification of their components.

Table 7 provides a comprehensive overview of biological inducers, detailing fermentation-based treatments and microbial derivatives, their optimal application parameters, mechanistic pathways, and quantitative results for bioactive compound accumulation during cereal and pseudocereal germination.

## 9. Combination of Inducers and Integrated Approaches

### 9.1. Synergies Between Physical and Biological Inducers

The combination of germination with ultrasonic treatment and fermentation in cereals, specifically wheat and barley, has been shown to have significant effects on the quality of the food ingredient obtained [3]. Regarding the enhancement of bioactive compounds, notable increases in the content of flavonoids and GABA were observed. Flavonoid content increased between 35% and 68%, being more pronounced in fermented wheat varieties. Similarly, GABA content increased significantly, between 300% and 400% in fermented ingredients compared to controls. Total antioxidant activity also experienced an increase, ranging between 31% and 51% [3].

The mechanism of action behind these improvements is attributed to the activation of enzymes during germination and fermentation, including proteolytic and amylolytic enzymes that improve digestibility [5,42]. Fermentation with a complex starter of microorganisms, such as *Streptococcus thermophilus*, *Lactobacillus* spp., and *Bifidobacterium* spp., contributes to the production of GABA from glutamic acid and increases the solubility and extractability of flavonoids [5]. Additionally, ultrasonic treatment before germination intensifies these processes, favoring the synthesis of bioactive compounds [3,10,11]. Therefore, the combination of germination with ultrasound and fermentation represents an effective strategy to obtain cereal food ingredients with higher bioactive compound content and antioxidant capacity, in addition to improving their uniformity and digestibility [3].

### 9.2. Synergies Between Physical and Chemical Inducers

The network analysis (Figure 1) provides mechanistic insights into these observed synergies. Physical inducers primarily connect to “growth” and “accumulation” bridge terms, while biological inducers demonstrate strong connectivity with metabolic process clusters. The success of combined physical-biological approaches can be interpreted through their complementary network positions: physical treatments create optimal cellular conditions (growth-mediated pathways), while biological inducers activate specific metabolic routes (secondary metabolite synthesis), resulting in enhanced bioactive accumulation through independent but complementary mechanisms.

The combination of physical and chemical inducers significantly modulates germination and enriches the bioactive profile of cereals and pseudocereals. In buckwheat and quinoa, the application of germination, along with pretreatments such as ultrasound, soaking, or the use of alkali, proved to be an effective strategy to increase the content of phenolic compounds and antioxidant activity [8]. Specifically, a substantial increase in AOA was observed in quinoa after 72 h of germination with ultrasound (64%) and alkali (53%), which was attributed to a greater accumulation of flavonoids and phenolic acids. US also favored the accumulation of important bioactive compounds such as rutin, quercetin, and gentisic acid in germinated quinoa, while soaking notably increased hesperidin content (58%).

Similarly, in corn grains, the combination of UV-B radiation and CaCl_2_ significantly elevated the concentration of CAROTS such as lutein and zeaxanthin compared to control samples. This increase was related to the regulation of key gene expression in the carotenoid biosynthesis pathway [69]. In selenium-enriched black rice, the use of ultrasound prior to hot air drying improved the extraction of phenolic compounds, with gallic acid being one of the most abundant, in addition to positively influencing the profile of volatile compounds, which could contribute to better preservation of grain quality [82].

Treatment with GA_3_ and KBC (potassium-enriched biochar) in wheat under osmotic stress increased chlorophyll *a* (up to 34.35%) and *b* (up to 9.09%) levels [83]. The general mechanism of action underlying these combined effects involves the activation of crucial metabolic pathways, such as the phenylpropanoid pathway, which is fundamental for the synthesis of phenolic compounds, flavonoids, and other bioactive metabolites [8]. Controlled germination, especially when combined with physical pretreatments such as ultrasound or chemicals, can induce moderate stress in seeds, which in turn activates endogenous defense systems, resulting in a greater accumulation of compounds with potent antioxidant activity [94].

The combined and strategic application of physical and chemical inducers during germination represents an effective methodology for selective enhancement of key bioactive compounds and a significant increase in antioxidant capacity in cereals and pseudocereals, highlighting their valuable potential as functional ingredients in the food industry.

### 9.3. Synergies Between Physical Inducers

The strategic combination of multiple physical inducers demonstrates enhanced effects beyond individual applications, evidencing true synergistic potential for bioactive compound optimization. The combined application of ultrasound (US) and PEF in wheat seedling juice resulted in the highest values of bioactive compounds and antioxidant activity compared to individual treatments, with total phenolic content increasing by 8.59%, total flavonoids by 14.06%, and chlorophyll by 12.06%, while antioxidant capacity measured by DPPH and ORAC increased by 8.58% and 2.34%, respectively [96]. This synergy suggests that PEF complements ultrasound action by enhancing cellular permeabilization and facilitating the extraction of intracellular components beyond what each treatment achieves individually.

The sequential combination of ultrasound and microwave (MW) as pretreatments in sorghum has demonstrated substantial bioactive compound accumulation, with ultrasound treatment (15 minutes) generating the highest GABA accumulation (87.14 μg/g) and achieving superior sprouting percentage (97.33%), while also elevating total phenolic content and antioxidant activity (DPPH inhibition reaching 84.53%) significantly higher than untreated controls [66]. Additionally, the combination of UV-B radiation with CaCl_2_ supplementation in yellow corn exhibited synergistic effects for carotenoid enhancement, with combined treatment significantly elevating lutein (+77.38%), zeaxanthin (+121.07%), *α*-cryptoxanthin (+75.19%), *β*-cryptoxanthin (+65.52%), *α*-carotene (+79.17%), and *β*-carotene (+86.49%) concentrations compared to individual treatments [69].

The underlying mechanisms involve complementary cellular modifications: ultrasound creates membrane alterations through cavitation that facilitate release and extraction of bioactive compounds while improving grain hydration through enhanced capillary flow [66,96], PEF induces electroporation, enhancing cellular permeability [96], while UV-B radiation stimulates biosynthetic pathways that are further optimized by mineral supplementation [69]. These synergistic combinations demonstrate that integrated physical approaches can achieve superior bioactive compound enhancement through multiple, complementary mechanisms of action, establishing a promising strategy to improve germination, enrich key bioactive compounds, and enhance antioxidant capacity in cereals and pseudocereals.

The integrated approaches discussed in the preceding sections, encompassing synergistic combinations between physical, chemical, and biological inducers, along with their processing conditions, reported synergies, and quantitative results for bioactive compound optimization, are comprehensively detailed in Table 8.

## 10. Applications in the Food Industry and Technological Considerations

The development of functional foods from germinated cereals and pseudocereals represents a promising biotechnological strategy for the development of functional foods with improved nutritional and bioactive properties. This natural process allows the transformation of food matrix components, increasing the bioavailability of nutrients and the synthesis of specific bioactive compounds (Table 9).

### 10.1. Functional Flours

Flours derived from germinated cereals and pseudocereals represent functional ingredients with versatile applications. Flours from germinated foxtail millet suitable for gluten-free foods and improved nutritional formulations have been developed [42]. It has been possible to obtain flour from germinated amaranth with higher protein and dietary fiber contents for products with increased nutritional value [50]. Flours from germinated millets with antidiabetic properties for formulations aimed at glycemic control have been obtained [93]. From germinated corn malted flours for gluten-free foods, natural sweeteners and enriched bakery products have been developed [2]. Buckwheat flour treated with high hydrostatic pressure for gluten-free bakery products with improved bioactive properties has been proposed [98]. Finally, flours from germinated quinoa and millets for food products with improved protein digestibility have been obtained [64].

### 10.2. Functional Bakery Products

Germinated cereals and pseudocereals offer excellent opportunities to develop functional bakery products. Wheat malt can be used as a “clean label” ingredient in baking, providing anti-aging capacity [44]. Germinated wheat ingredients have been developed to improve the nutritional quality of baked products [10]. Cookies with sorghum sprouts with antioxidants and anti-inflammatory properties stable after baking have been made [49]. Similarly, germinated barley ingredients with prebiotic potential for bakery products have been created [52]. Studies proposed germinated rice malt for baked products with improved functional properties [51].

### 10.3. Functional Breakfast Cereals and Snacks

Breakfast cereals incorporating germinated grains provide an effective vehicle for delivering bioactive compounds through daily dietary consumption. Cereals formulated from germinated wheat, barley, and sorghum have been developed specifically to reduce chronic disease risk through enhanced phenolic content and antioxidant capacity [53]. Amaranth sprouts demonstrate significant potential as functional ingredients in breakfast formulations due to their superior antioxidant properties [80]. Germinated blue corn flour has been successfully applied in breakfast cereal development, exhibiting pronounced hypoglycemic properties that support glycemic management [92]. Additionally, breakfast cereals enriched with GABA from germinated barley and rye (*Secale cereale* L.) offer therapeutic benefits for individuals with diabetes or elevated colon cancer risk [27].

Snack products derived from germinated cereals and pseudocereals represent innovative functional food alternatives with targeted health benefits. Specialized snacks formulated from germinated amaranth, quinoa, and buckwheat have been developed to address the nutritional needs of individuals with celiac disease while providing enhanced bioactive compound profiles [1]. Germinated buckwheat-based snacks demonstrate cytoprotective efficacy against oxidative cellular damage, offering potential applications in preventive nutrition [33]. Functional snack formulations utilizing germinated buckwheat and quinoa as primary sources of phenolic compounds have shown promise for antioxidant-enhanced convenience foods [8]. Furthermore, amaranth sprout-based snacks provide superior bioactive compound concentrations, establishing their potential as premium health-oriented snack alternatives [80].

The potential applications of cereals and pseudocereals processed using enhanced germination techniques with emerging inducers, along with their recommended inducers, ideal matrices, development status, and commercial prospects, are systematically presented in Table 9.

### 10.4. Functional Beverages

Beverages derived from germinated cereals and pseudocereals represent an innovative delivery system for bioactive compound consumption with enhanced bioavailability and functional properties. Wheat seedling juices have been developed with extended shelf-life characteristics, positioning them as viable supplements and functional beverages for commercial applications [96]. Non-dairy probiotic beverage formulations incorporating germinated grains combined with plant-based milk alternatives have been specifically designed to address the nutritional needs of lactose-intolerant consumers while delivering enhanced bioactive profiles [55].

Functional juice preparations from germinated wheat demonstrate diverse therapeutic properties, including anti-inflammatory and immunomodulatory activities that support immune system regulation [37]. Additionally, wheat-derived beverages exhibit antihypertensive and neuroprotective properties, offering potential applications in cardiovascular health management and cognitive function support [10]. These developments establish germinated cereal-based beverages as promising functional food platforms that combine convenience with targeted health benefits through optimized bioactive compound delivery systems.

### 10.5. Fermented Foods

Fermented foods based on germinated cereals and pseudocereals offer amplified functional benefits. Fermented products from germinated corn with a prebiotic effect to address nutritional deficiencies in vulnerable populations have been developed [4]. The elaboration of fermented products from germinated sorghum and millet with probiotic properties for functional foods and infant nutritional formulations is reported [24]. From fermentation with *Rhizopus oligosporus* in germinated djulis, food supplements and nutraceutical ingredients have been produced [41]. Germinated and fermented barley products with high GABA and *β*-glucan content as functional components have been formulated [5]. Fermented products from germinated blue corn have been developed for foods with nutraceutical properties [50], and germinated barley malt has been applied to produce fermented beverages with prebiotic properties [107].

### 10.6. Bioactive Concentrates

Bioactive concentrates derived from germinated cereals and pseudocereals offer applications as functional ingredients. Concentrates from germinated barley rich in saponarin, GABA, and policosanols have been developed as ingredients with antioxidant and neuroprotective properties [70]. Concentrates from germinated buckwheat with rutin, quercetin, and epicatechin have been obtained for the food and cosmetic industry [112]. Germinated wild rice (*Zizania latifolia*) concentrates have been used as natural nutrient enhancers [76]. From germinated bitter buckwheat, concentrates with high GABA content have been developed for foods with antihypertensive properties [30].

### 10.7. Functional Foods for Glycemic Control

Various researchers have developed foods for glycemic control from germinated cereals and pseudocereals. Products with germinated millets with digestive enzyme inhibition for the management of postprandial hyperglycemia have been formulated [93]. Foods from germinated quinoa with a lower glycemic index for people with obesity and type 2 diabetes have been created [100]. Products with germinated djulis with dipeptidyl peptidase IV (DPP-IV) inhibitory properties have been developed [41]. Likewise, germinated quinoa products with *α*-amylase and *α*-glucosidase inhibition have been proposed [48]. Finally, germinated blue corn foods with hypoglycemic properties have been elaborated [92].

### 10.8. Infant Foods

Germinated cereals and pseudocereals offer ideal characteristics for improved infant foods. Germinated amaranth flours for infant foods with better essential amino acid profile have been developed [50]. Formulations of germinated kodo millet for weaning foods and porridges with low viscosity and higher GABA content have been proposed [77]. Preparations of germinated and fermented corn with better bioavailability of iron and zinc for infants have also been obtained [4]. It is also possible to formulate porridges and complementary foods with improved digestibility [2], developing infant nutritional formulations with germinated and fermented sorghum and millet [24].

### 10.9. Foods with Improved Bioavailability

Foods from germinated cereals and pseudocereals present improved bioavailability of essential nutrients. For this purpose, germinated corn products with greater bioavailability of iron and zinc through reduction of phytates can be created [4]. From brown rice germinated in acidic medium, products with improved bioavailability of calcium and zinc can be developed [38]. Foods with ultrasound-treated germinated brown rice with greater bioavailability of iron and calcium have been formulated [31]. Iron-enriched germinated buckwheat products for populations with deficiencies of this mineral have been made [91]. Germinated and fermented sorghum and millet foods with better mineral bioavailability have been proposed [24].

### 10.10. Functional Malted Products

Several studies specifically address malted products with functional properties. Barley malt with improved prebiotic properties has been developed [107]. Malt with reduced germination times, yet preserving its prebiotic potential, has been developed [52]. Rice malt with improved antioxidant properties has been developed [51]. Malts of various cereals have been reported to provide products with greater stability during processing [53].

Germinated cereals and pseudocereals represent an exceptional raw material for developing a diverse range of functional foods, including flour, bakery products, breakfast cereals, beverages, and snacks, as well as bioactive concentrates and products tailored to specific nutritional needs. The diversity of functional properties enables these products to be tailored to specific health needs, constituting a promising field for the food industry and the development of preventive nutritional strategies.

## 11. Bioavailability and Biological Efficacy of Enhanced Bioactive Compounds

The bioavailability and physiological efficacy of bioactive compounds in germinated cereals and pseudocereals show promising results, yet significant methodological limitations constrain clinical translation.

Mineral bioavailability demonstrates consistent improvements through phytase activation, with phytate reductions of 85.6–90.1% and mineral bioaccessibility increases of 32.9–44.4% for calcium and zinc [1,4,24,38]. However, these findings rely exclusively on in vitro models that may not reflect human gastrointestinal conditions, and no human bioavailability studies validate these predictions.

Phenolic compound bioaccessibility studies show enhanced release during simulated digestion and improved protein digestibility exceeding 82% [22,42,48,49]. Yet methodological inconsistencies in gastrointestinal simulation protocols limit comparative analysis, and the correlation between in vitro digestibility and actual nutritional absorption remains unestablished.

In vivo evidence, while compelling, remains limited. The single rat study demonstrating improved oxidative stress biomarkers [119] requires replication, while cellular studies showing cytoprotective effects utilize concentrations potentially unachievable through dietary consumption [33,49,92].

Enzymatic inhibition studies demonstrate reproducible effects, including 650% *α*-amylase inhibition in barley and 135% ACE inhibition in buckwheat [27,30,41,93]. However, the clinical relevance of these concentrations through normal dietary intake remains unclear, and supraphysiological effects may interfere with normal digestion.

Gut microbiota modulation shows prebiotic effects in vitro [34,48,52], yet these findings may not reflect human gut microbiome complexity.

Critical knowledge gaps include (1) absence of human clinical trials, (2) lack of dose–response relationships, (3) insufficient understanding of inter-individual variability, (4) limited safety data for enhanced consumption, and (5) inadequate food matrix considerations. While mechanistically sound, the evidence requires substantial clinical validation before functional food health claims can be substantiated.

## 12. Challenges and Technological Considerations

The industrial implementation of functional foods derived from germinated cereals and pseudocereals presents multiple technical challenges that require a systematic approach to achieve commercially viable products.

The optimization of germination conditions constitutes a fundamental challenge. The need for precise control in the intensity and duration of exposure to physical inducers to prevent inhibitory effects on germination and seedling development has been documented [43]. In accordance, it has been demonstrated that rigorous control of exposure time to UV-B radiation is critical to maximize the synthesis of bioactive compounds without compromising vegetative development [26]. The complexity of balancing the induction of bioactive compounds through salt stress against the inevitable inhibition of growth was identified, quantifying a 60% reduction in sprout length at 300 mM NaCl concentrations [89].

Industrial scaling represents a significant technological barrier. Technical incompatibilities when transferring optimal laboratory conditions to commercial production systems have been documented, where the available light intensity (maximum 200 lux) was substantially lower than that experimentally determined as optimal (6000 lux) [47]. The need for specific parametric optimization according to the cereal matrix has been established, with critical dependence on initial humidity and electrical conductivity for the effectiveness of pulsed electric fields at an industrial scale [44]. The criticality of temporal control in ozone exposure has been identified, given that prolonged periods (>6 h) induce significant degradation of bioactive compounds [19].

The stability of compounds during processing and storage constitutes a determining technological limitation. It has been evidenced that certain bioactive compounds reach maximum concentration at specific temporal points during germination to subsequently decrease, indicating the need for precise determination of the optimal harvest time [16]. Instability in anthocyanins and aromatic compounds such as 2-acetyl-1-pyrroline during extended germination (3–4 days) has been identified, underlining the importance of strict control of process times [90]. It is reported that UV-B radiation significantly inhibits sprout length and germination percentage, although this inhibition is partially attenuated through supplementation with CaCl_2_ [69,87].

Microbiological control during germination represents a critical safety concern. Technical limitations in the treatment of large volumes of seeds with plasma have been documented, as well as variable responses according to the cereal species [46]. There is evidence that plasma treatment for the reduction of mycotoxins, such as deoxynivalenol, does not achieve complete elimination (maximum 58.4% reduction) due to insufficient penetration of reactive species into the inner layers of the grain where mycotoxins persist [45]. Restrictions in the efficacy of microbial inactivation through UV-C in seeds with irregular surfaces have been identified, characterizing a “shadow effect” that compromises the effectiveness of the treatment [28].

Sensory and organoleptic properties present significant challenges for commercial acceptability. The potential degradation of key aromatic compounds (2-acetyl-1-pyrroline) during treatment with plasma-activated water has been documented [4]. The need for specific validation according to geographic context and rigorous evaluation of sensory acceptability of fermented products for different cultural environments has been established [37]. Methodological deficiencies in the comprehensive evaluation of sensory attributes such as flavor, texture, and aroma that decisively determine consumer acceptability have also been noted [2].

Specific limitations according to the inducer applied. It has been observed that prolonged durations of ultrasonic treatment (>15 min) reduce the efficacy in GABA accumulation and can cause excessive biomass loss [22]. It has been determined that after 48 h, germination with slightly acidic electrolyzed water manifested adverse effects on the germinative potential of brown rice [73]. Similarly, exposure to high NaCl concentrations significantly reduces sprout length (21% reduction) and germination percentage (from 85% to 55%) [87].

The industrialization of functional foods derived from germinated cereals and pseudocereals requires systematically addressing multiple technological challenges related to optimization of processing parameters, industrial scaling, stability of bioactive compounds, microbiological control, and sensory properties. Resolving these limitations is critical for the successful development of commercially viable products with preserved functional properties throughout their shelf life.

The bioavailability and efficacy of bioactive compounds present in germinated cereals and pseudocereals constitute critical parameters for determining their functional value as ingredients in food matrices. Contemporary research ranges from in vitro digestibility models to evaluations of specific biological activities, providing substantial evidence on the potential of these compounds to confer specific physiological benefits.

Multiple investigations have systematically evaluated mineral bioavailability through in vitro digestion models. It has been demonstrated that the sequential integration of soaking, germination, and fermentation with Lactobacillus plantarum in corn matrices significantly reduces phytate concentration (85.6%), optimizing the bioavailability of iron and zinc by decreasing the molar phytate ratios (81%, from 40.76 to 7.77) and phytate (85%, from 41.42 to 6.24) [4]. Mineral bioaccessibility in brown rice germinated under acidic conditions has been quantified, documenting substantial increases in bioavailability of calcium (32.9%, from 18.84% to 25.04%) and zinc (44.4%, from 19.56% to 28.24%) [38]. Synergistic effects between germination and spontaneous fermentation have been observed, achieving significant reductions of phytates (90.1% in sorghum and 85.1% in millet), with the consequent improvement in bioavailability of iron, zinc, and calcium [24].

Research has extensively characterized phenolic compound stability and bioaccessibility. Studies evaluated phenolic compound bioaccessibility in sorghum sprouts treated with UV-A radiation incorporated in cookie matrices, verifying that these compounds maintain stability during thermal processing and preserve their bioaccessibility after simulated gastrointestinal digestion [49]. Research analyzed germination protocol influence on phenolic compound bioaccessibility in quinoa, documenting significant increases in release and transport rate during in vitro digestion [48]. Studies meticulously characterized bioaccessibility of 47 specific phenolic compounds in germinated quinoa, evidencing significant increases in the bioavailable fraction of determined phenolic acids and flavonoids [16].

Regarding biological efficacy, various studies have quantitatively evaluated antioxidant activity through complementary methodologies. The antioxidant capacity of brown rice germinated in slightly acidic electrolyzed water has been quantified through DPPH, ABTS, and FRAP assays, recording increases of 839%, 792%, and 934%, respectively [25]. An extraordinary increase (3700%) in antioxidant activity determined by DPPH in quinoa sprouts subjected to controlled salt stress has been evidenced [89]. The cytoprotective effect against oxidative damage has been evaluated, demonstrating that extracts of buckwheat sprouts treated with sucrose and calcium conferred significant protection to human liver cells (HepG2) and dermal fibroblasts (Hs68) against experimentally induced oxidative stress [33].

Research has rigorously characterized enzymatic inhibition related to carbohydrate metabolism. Studies determined that secondary metabolites produced during germination and fermentation of djulis exhibit significant inhibitory activity on dipeptidyl peptidase-IV (DPP-IV) and angiotensin-converting enzyme (ACE), critical biomarkers for glycemic control and blood pressure regulation, respectively [41]. Research quantified inhibitory activity of germinated millet extracts on *α*-amylase and *α*-glucosidase, identifying potent inhibitory effects relevant for attenuating postprandial hyperglycemia [93]. Studies documented that *α*-glucosidase inhibitory activity in germinated sorghum increased by 25% (from 16% to 20%), while *α*-amylase inhibition in germinated barley increased by 650% (from 3% to 35%) [27].

Antihypertensive capacity has been the subject of specific characterization. The inhibitory activity on angiotensin-converting enzyme (ACE) in bitter buckwheat sprouts treated with pyridoxal phosphate has been quantified, evidencing an increase of 135% (from 32.86% to 77.26%) in this activity, suggesting potential application in blood pressure regulation [30]. Research implemented in vivo models with rats subjected to oxidative stress, demonstrating that extracts of germinated quinoa sprouts significantly improved oxidative stress biomarkers, including malondialdehyde (MDA), glutathione (GSH), and superoxide dismutase (SOD) [119].

Intestinal microbiota modulation represents an additional functional parameter evaluated. The prebiotic effect of germinated quinoa has been characterized, evidencing its capacity to enhance the production of short-chain fatty acids and favorably modulate colonic microbiota composition [48]. The prebiotic potential of germinated barley malt has been specifically evaluated, documenting selective stimulation of the growth of probiotic bifidobacteria without promoting the proliferation of potentially pathogenic microorganisms [52].

Various studies have addressed bioavailability through characterization of morphostructural modifications in food components. In vitro digestibility studies have been integrated with scanning electron microscopy analysis, demonstrating that ultrasound treatment significantly alters the supramolecular structure of starch in germinated brown rice, increasing its susceptibility to enzymatic hydrolysis and optimizing the bioavailability of various nutrients [31]. It has been evidenced that germination preferentially affects the molecular structure of amylose, while amylopectin conformation remains relatively stable, generating specific modifications in starch digestion kinetics with direct implications for postprandial glycemic response [72,100].

The available scientific evidence conclusively demonstrates that germination processes, particularly when integrated with specific inducers, significantly optimize the bioavailability and biological efficacy of various bioactive compounds present in cereals and pseudocereals. The underlying mechanisms include reduction of antinutritional factors, structural modifications that favor compound release, and biochemical transformations that enhance specific biological activity. These findings support the potential of germinated cereals and pseudocereals as functional ingredients with demonstrable physiological benefits, although validation through controlled clinical studies in humans is required to fully confirm these effects under habitual consumption conditions.

## 13. Conclusions and Future Perspectives

The comprehensive review of emerging inducers for cereal and pseudocereal germination establishes a theoretical–practical framework demonstrating that physical, chemical, and biological inducers can significantly enhance bioactive compound content through defined mechanisms of action. Co-occurrence network analysis revealed strategic research opportunities based on bridge terms connecting multiple thematic clusters, with “growth” (146 links), “GABA” (97 links), and “accumulation” (101 links) emerging as key connectors that suggest priority areas for mechanistic convergence studies and species-specific optimization approaches.

Physical inducers, particularly ultraviolet radiation, electromagnetic fields, ultrasound, and cold plasmas demonstrated efficacy for enhancing GABA, phenolics, flavonoids, and carotenoids, though parameter optimization remains critical for balancing bioactive maximization with vegetative viability. Chemical inducers, including phytohormones and stress-inducing compounds, act through moderate stress simulation that stimulates secondary metabolic pathways, while biological inducers offer synergistic advantages by combining bioactive enhancement with antinutritional factor reduction. Synergistic combinations between inducer categories consistently outperform individual applications by targeting different network nodes simultaneously, creating additive effects across interconnected pathways.

Industrial implementation faces critical challenges, including scalability, compound stability, microbiological control, and sensory acceptability, that require systematic technological development. While in vitro and in vivo studies confirm significant improvements in antioxidant capacity and bioactive properties, clinical validation in humans under realistic consumption conditions remains essential for substantiating health claims and establishing commercial viability.

Future research priorities encompass industrial scalability through economically viable preservation technologies, clinical validation establishing dose–response relationships, mechanistic elucidation enabling predictive modeling across species, and sustainability assessment incorporating comprehensive lifecycle evaluation. Success requires multidisciplinary collaboration integrating basic science, food technology, clinical nutrition, and market studies to develop products that combine nutritional excellence with demonstrated health benefits, positioning germinated cereals as a mature field ready for industrial application and clinical validation.

## Figures and Tables

**Table 1:** Bioactive compounds induced during germination of cereals and pseudocereals: characteristics, benefits, mechanisms of action, and scientific evidence.

Group	Characteristics and Benefits	Bioactive Compounds	Mechanism of Biological Action	Matrices Studied	Ref.
Phenolic compounds	Potent antioxidants that neutralize free radicals, reduce lipid peroxidation, prevent cellular oxidative damage, reduce chronic disease risk, and have anti-inflammatory effects.	PHEA: *p*-HBA, CHLA, ELLA, SALA, *p*-COU, GENT, FERA. FLVN: RUTI, QUER, KAEM, CATCH, EPIC. ANTH and other POLY including TAN.	Act as free radical scavengers, chelate pro-oxidant metals, modulate inflammatory pathways, inhibit oxidative enzymes, and protect cell membranes.	Red sorghum, pearl millet, djulis (*Chenopodium formosanum*), naked barley (*Hordeum vulgare* L. var nudum), blue corn, foxtail millet, wheat (*Triticum aestivum* L.), barley, buckwheat, quinoa.	[3,5,8,24,41,42,50]
Neurotransmitters	Main inhibitory neurotransmitter in CNS. Related to hypotensive, antidepressant, and nervous system regulatory effects. Improves sleep, reduces anxiety, regulates blood pressure.	GABA and its precursors such as glutamic acid.	Functions as an inhibitory neurotransmitter, modulates neuronal excitability, reduces neurotransmitter release, and exerts a calming effect.	Soft wheat, barley, naked barley, djulis, rice (*Oryza sativa* L.), buckwheat, finger millet (*Eleusine coracana* (L.) Gaertn.), and sorghum.	[3,5,15,41,76,77,78]
Bioactive peptides	Possess antioxidant, antihypertensive, antimicrobial, immunomodulatory, and antithrombotic activity. Improve mineral bioavailability and may have hypocholesterolemic effects.	AABA, BIOP with antioxidant activity, antimicrobial peptides, oligopeptides and FAA, immunomodulatory peptides.	Inhibit key enzymes in physiological processes, interact with opioid receptors, neutralize free radicals, and bind to minerals, increasing their bioavailability.	Rice, djulis, corn, buckwheat, wheat, quinoa.	[6,37,41,51,67,69,79]
Melatonin and indolic compounds	Melatonin is a potent antioxidant and circadian rhythm regulator. INDO has neuroprotective and anti-inflammatory activity. Improve sleep, protect neurons, modulate the immune system.	MELA, TRYP, *p*-CQA, FERQ.	MELA neutralizes free radicals in lipophilic and hydrophilic environments. INDO acts as a neurotransmitter precursor, modulates immune pathways.	Amaranth (*Amaranthus* spp.)	[80]
Vitamins	Improves nutritional profile and biological value, helps combat micronutrient deficiencies, especially in vulnerable populations.	VITB: THIA, RIBO, NIAC. ASCA and VITA precursors. FOL.	Reduces antinutritional compounds, transforms inactive vitamin forms to active forms, and increases solubility and stability.	Corn, sorghum, pearl millet, barley, blue corn, foxtail millet.	[4,5,24,41,42,50]
Antioxidant enzymes	Contribute to the detoxification of reactive oxygen species, reducing oxidative stress. Help prevent chronic diseases related to oxidative damage.	SOD, CAT, POD, GPx, GR.	Dismutation of superoxide anion, degradation of hydrogen peroxide, reduction of hydroperoxides, and maintenance of the antioxidant cycle.	Wheat, buckwheat, quinoa, corn, millet, barley, rice.	[8,32,37,69,81,82,83]
Various phytochemicals	Possess antioxidant, anti-inflammatory, anticarcinogenic, and immune system modulating activity. Contribute to chronic disease prevention, benefit visual and cardiovascular health.	CAROT, CHL-a, CHL-b, TERP, TERD, SAPN, natural pigments, PHYS.	Neutralize free radicals, modulate inflammation pathways, protect cell membranes, photosensitize activity, and induce apoptosis in tumor cells.	Corn, djulis, millet, rice, barley.	[6,32,37,41,69,83,84]
Dietary fiber	Contributes to gastrointestinal health, prebiotic effect, cholesterol reduction, glycemic control, and satiety sensation. It prevents cardiovascular diseases, type 2 diabetes, and certain cancers.	*β*-GLU, ARBX, OLIGS, SOLDF, INDF, REST.	Increases intestinal viscosity, ferments via microbiota producing short-chain fatty acids, binds bile acids, stimulates beneficial bacteria growth.	Barley, wheat, sorghum, millet.	[5,10,27,85]

Note: This table presents a classification of the main groups of bioactive compounds generated during the germination of cereals and pseudocereals, their characteristics, mechanisms of biological action, and the scientific evidence supporting their benefits. The references correspond to research conducted between 2015 between 2025 on various cereals and pseudocereals, highlighting the nutraceutical potential of these bioactive compounds for human health. Abbreviations: AABA, Angiotensin-Converting Enzyme (ACE) inhibitory peptides; ANTH, Anthocyanins; ARBX, Arabinoxylans; ASCA, Ascorbic acid (Vitamin C); *β*-GLU, *β*-Glucan; BIOP, Bioactive peptides; CAROT, Carotenoids; CAT, Catalase; CATCH, Catechin; CHL-*a*, Chlorophyll *a*; CHL-*b*, Chlorophyll *b*; CHLA, Chlorogenic acid; CNS, Central Nervous System; ELLA, Ellagic acid; EPIC, (-)-Epicatechin; FAA, Free amino acids; FERA, Ferulic acid; FERQ, Feruloylquinic acid; FLVN, Flavonoids; FOL, Folates; GABA, *γ*-Aminobutyric acid; GENT, Gentisic acid; GPx, Glutathione peroxidase; GR, Glutathione reductase; INDF, Insoluble dietary fiber; INDO, Indolic compounds; KAEM, Kaempferol; MELA, Melatonin (N-acetyl-5-methoxytryptamine); NIAC, Niacin (Vitamin B3); OLIGS, Oligosaccharides; *p*-COU, *p*-Coumaric acid; *p*-CQA, *p*-Coumaroylquinic acid; *p*-HBA, *p*-Hydroxybenzoic acid; PHEA, Phenolic acids; PHYS, Phytosterols; POD, Peroxidase; POLY, Polyphenols; QUER, Quercetin; REST, Resistant starch; RIBO, Riboflavin (Vitamin B2); RUTI, Rutin; SALA, Salicylic acid; SAPN, Saponins; SOD, Superoxide dismutase; SOLDF, Soluble dietary fiber; TAN, Tannins; TERD, Terpenoids; TERP, Terpenes; THIA, Thiamine (Vitamin B1); TRYP, Tryptophan; VITA, Vitamin A precursors; VITB, Vitamin B complex.

**Table 2:** Physical inducers for germination of cereals and pseudocereals I: optimization of controlled germination in quinoa (*Chenopodium quinoa* Willd.) for maximization of bioactive compounds.

Species	Optimal Processing Parameters	Measurement Basis	Bioactive Compounds Analyzed and Quantitative Results	Ref.
Quinoa	22 °C, 80% RH, 144 h; 48 h dark then 16/8 h light/dark	Dry basis	5-MTHF: +8567% (0.203 → 17.47 mg/100 g). TPC: +397% (2.62 → 10.42 mg GAE/g). TFC: +325% (1.65 → 5.36 mg CE/g). ANTH: +958% (1.7 → 16.3 μg CE/g). LUT: +19787% (0.53 → 105.1 mg/100 g). ASCA: +276% (8.25 → 22.82 mg/100 g). RIBO: +728% (0.061 → 0.444 mg/100 g)	[20]
Quinoa	25 °C, 48 h, light treatment, 95% RH	Dry basis	TPC: Gray quinoa +50% (0.128 → 0.192 mg GAE/g), White quinoa +16.4% (0.128 → 0.149 mg GAE/g), Black quinoa +23.4% (0.141 → 0.173 mg GAE/g)	[118]
Quinoa	72–120 h at room temperature, 4 h soaking, 16/8 h light/dark	Dry basis	TPC: +35% (0.191 → 0.258 mg GAE/g). RUT: +245% (23.71 → 81.8 μg/g). KAEM: +760% (2.83 → 24.37 μg/g). QUER: +8333% (0.18 → 15.18 μg/g). PINO: +933% (1.65 → 17.05 μg/g). ISOR: +1250% (0.36 → 4.86 μg/g). AOA: variable increases	[115]
Quinoa	25 °C, 36–72 h, 90–95% RH, darkness	Not specified	GABA: +117% (52.6 → 114.2 mg/100 g). FPHE: +32% (2.67 → 3.51 mg GAE/g). FLVN: +81% (0.787 → 1.425 mg CE/g). BPHE: +22% (2.89 → 3.54 mg GAE/g). BFLVN: +127% (0.453 → 1.028 mg CE/g). FERA: +104% (162.16 → 331.32 μg/g). SALA: +597% (15.37 → 107.12 μg/g). KAEM: +1331% (2.05 → 29.35 μg/g). AOA: +1722% (6.25 → 113.81 μmol TE/g)	[16]
Quinoa	25 °C, 72 h, 95% RH, followed by 40 °C drying	Dry basis	TPC: White +74.4% (0.259 → 0.452 mg GAE/g), Red +60.9% (0.275 → 0.443 mg GAE/g), Black +43.3% (0.327 → 0.469 mg GAE/g). TFC: White +95.4% (0.633 → 1.238 mg CE/g). AOA: White +99.6% (45.32 → 90.48 μmol TE/g), Black +100.8% (50.36 → 101.12 μmol TE/g)	[23]
Quinoa	25 °C, 8 h soaking (1:10), germination to 1 cm sprouts	Dry basis	TPC: +21.91%. FERA: +289.11% (74.4 → 289.5 μg/g). Q3OG: +55.11% (722.6 → 1120.8 μg/g). QUER: +75.21% (48.0 → 84.1 μg/g). GACA: +79.85% (40.2 → 72.3 μg/g)	[48]
Quinoa	20 °C, 96 h darkness, watering each 12 h	Dry basis	TPC: White +50% (0.128 → 0.192 mg GAE/g), Red +13.7% (0.161 → 0.183 mg GAE/g), Black +22.7% (0.141 → 0.173 mg GAE/g)	[100]
White quinoa	28 °C, 48 h, periodic water spraying	Dry basis	TPC: Foxtail millet +36% (2.50 → 2.89 mg GAE/g), Proso millet +15% (0.86 → 1.16 mg GAE/g), White quinoa +24% (1.04 → 1.29 mg GAE/g). TFC: Foxtail millet +4.7% (6.28 → 6.57 mg CE/g), Proso millet +25.4% (0.62 → 0.78 mg CE/g), White quinoa +23.5% (0.70 → 0.86 mg CE/g)	[64]
Red and yellow quinoa	17 ± 1 °C, 90% RH, darkness, 6 days	Wet basis	TPC: Red +178.9% (1.05 → 2.93 mg GAE/g), Yellow +130.4% (1.12 → 2.59 mg GAE/g). AOA: Red +69.8% (174 → 295.6 μmol TE/g), Yellow +75.3% (120 → 210.4 μmol TE/g). FERA: Red +114.6% (173.3 → 371.9 μg/g), Yellow +47.2% (551.4 → 811.5 μg/g). TFC: Red +47.2% (0.71 → 1.04 mg CE/g), Yellow +165.4% (0.90 → 2.40 mg CE/g)	[119]
Red and white quinoa	20 °C, 4 days darkness, drying at 30 °C	Dry basis	TPC: Red +105% (1.36 → 2.79 mg GAE/g), White +105% (0.72 → 1.48 mg GAE/g). VANA: Red +9242% (1.4 → 130.8 μg/g). FERA: Red +367% (18.9 → 88.2 μg/g). AOA: Red +50% vs. White	[29]
Quinoa	20 °C, 42 h	Dry basis	TPC: +84.2% (2.71 → 4.99 mg GAE/g). GABA: +445.7% (22.41 → 122.32 mg/100g). AOA: +30% (43.43 → 56.42 μmol TE/g)	[117]
Quinoa	4 °C 24 h, 10 °C 72 h, 16/8 h photoperiod, >10,000 lx	Not specified	SAPN: CQE_01 −60% (4.2 → 1.7 mg/g), CQE_02 +80% (1.7 → 3.1 mg/g), CQE_03 no significant changes	[84]

Note. This table summarizes the findings of different studies on the effect of controlled germination, as a physical inductor, on the bioactive compound content of quinoa. Abbreviations: 5-MTHF, 5-Methyltetrahydrofolate; ANTH, Anthocyanins; AOA, Antioxidant activity; ASCA, Ascorbic acid (Vitamin C); BFLVN, Bound flavonoids; BPHE, Bound phenolic compounds; CE, Catechin equivalents; CQE, *Chenopodium quinoa* extracts; FERA, Ferulic acid; FLVN, Flavonoids; FPHE, Free phenolic compounds; GABA, *γ*-Aminobutyric acid; GACA, Gallic acid; GAE, Gallic acid equivalents; ISOR, Isorhamnetin; KAEM, Kaempferol; LUT, Lutein; PINO, Pinocembrin; Q3OG, Quercetin-3-O-glucoside; QUER, Quercetin; RIBO, Riboflavin (Vitamin B2); RUT, Rutin; SALA, Salicylic acid; SAPN, Saponins; TE, Trolox equivalents; TFC, Total flavonoid content; TPC, Total phenolic content; VANA, Vanillic acid.

**Table 3:** Physical inducers for germination of cereals and pseudocereals II: optimal conditions of temperature, humidity, and time (controlled germination) for the accumulation of bioactive compounds.

Species Studied	Optimal Parameters	Measurement Basis	Bioactive Compounds and Quantitative Results	**Ref.**
Buckwheat	25 °C, 72 h	Not specified	TFC: +53.2% (6.51 → 9.97 mg CE/g, cv. Pintian), +49.3% (5.59 → 8.34 mg CE/g, cv. Suqiao); TPC: +30% (values not reported, 72 h); AOA: significant increase at 3 days	[12]
Amaranth, quinoa, buckwheat	25 °C, 72 h, soaking 16 h	Dry basis	TPC: amaranth +126.62% (0.33 → 0.74 mg GAE/g), buckwheat +125.32% (2.10 → 4.74 mg GAE/g), quinoa +71.56% (0.48 → 0.82 mg GAE/g); AOA: buckwheat +178.38% (31.69 → 88.22%), amaranth +87.47% (18.75 → 35.15%), quinoa +34.88% (46.41 → 62.60%)	[1]
Amaranth	35.86 °C, 22 h	Not specified	AOA: +43.8% (10.23 → 14.71%); TPC: +4.5% (0.47 → 0.49 mg GAE/g); TFC: +3.2% (0.069 → 0.071 mg CE/g); OLEA: +8.2% (1.84 → 1.99%); LINA: +18.6% (1.94 → 2.30%)	[50]
Amaranth	28 ± 2 °C, 72 h darkness, 30 min 0.2% formaldehyde pretreatment	Dry basis	TPC: +52.7% (1.08 → 1.65 mg TAE/g); TFC: +33.0% (0.24 → 0.32 mg RE/g); FERA: +28.0% (0.11 → 0.15 mg/g); *p*-HBA: +19.4% (0.11 → 0.13 mg/g); AOA: +54.3% (0.32 → 0.49 mg TE/g)	[65]
Rice, Corn	30–35 °C, 192 h, 12 h initial soaking, watering every 12 h	Not specified	TPC: corn +600% (0.070 → 0.419 mg GAE/g), rice +10% (0.173 → 0.192 mg GAE/g); TFC: corn +230% (0.36 → 1.16 mg QE/g), rice +74% (0.34 → 0.60 mg QE/g); AOA FRAP: corn +348% (5.56 → 24.90 μmol FeSO_4_/g), rice +27% (20.55 → 26.06 μmol FeSO_4_/g)	[18]
Chinese wild rice	30 °C darkness, 120 h	Not specified	TPC: +96.6% (1.07 → 2.10 mg GAE/g); FERA: +75% (0.49 → 0.85 mg/g); CATCH: +67.3% (0.38 → 0.64 mg/g); GABA: +729% (0.076 → 1.47 mg/g); AOA: +50% (spectrophotometric evaluation)	[76]
Oat	20 °C, 48–72 h, >85% RH, 24 h soaking	Not specified	Total AVEN: +29,300% (0.006 → 1.76 mg/g); AVN 2c: +1993–2130% (0.014 → 0.28–0.31 mg/g); AVN 2p: +900% (0.012 → 0.12 mg/g); AVN 2f: +1100% (0.009 → 0.11 mg/g); AVN-hexosides: +2,100% (0.001 → 0.022 mg/g)	[35]
Hulled oat, dehulled oat	16 °C 216 h (Barra), 18 °C 156 h (Meeri)	Dry basis	FPHE: Barra +63.7% (2.27 → 3.72 mg GAE/g), Meeri +165.8% (2.22 → 5.89 mg GAE/g); *β*-GLU: Barra −46.8% (20.3 → 10.8 g/kg), Meeri −55.9% (34.5 → 15.2 g/kg); AOA: Barra +172.2% (5.75 → 15.64 mg TE/g), Meeri +369.5% (5.38 → 25.25 mg TE/g)	[85]
Barley	22 °C, 240 h, *ad libitum* irrigation, 13.48–19.98 cm height	Dry basis	POLY: +49.8% (25.3 → 37.9 mg QE/g); PHEA: +41.0% (6.30 → 8.88 mg CAE/g); AOA DPPH: +175.0% (4.08 → 11.22 mg TE/g); AOA ABTS: +127.3% (5.42 → 12.32 mg TE/g); AChE inhibition: +610.0% (0.10 → 0.71 μM Es/g)	[102]
Barley	25 °C, 72 h for phenolics, 20 °C, 35 h for prebiotics	Dry basis	TPC: +61.8% (0.091 → 0.148 mg GAE/g); FERA: +89.1% (0.028 → 0.052 mg/g); *p*-COU: +127.3% (0.005 → 0.012 mg/g); GACA: +91.8% (0.018 → 0.034 mg/g)	[52]
Barley, Tibetan barley, rice	30 °C, 96 h barley/Tibetan barley, 48 h rice, soaking 8 h at 30 °C, drying 55 ± 5 °C	Dry basis	TPC: +23–41% (values not specified); AOA: +36–64% (values not specified)	[55]
Naked barley	25 °C, 36 h, infrared drying (600 W/m^2^, 20 °C)	Not specified	VITX: +386% (0.40 → 1.91 mg/g); RUTI: +379% (0.005 → 0.023 mg/g); HESP: +775% (0.002 → 0.016 mg/g); FERA: +766% (0.002 → 0.019 mg/g)	[14]
*Chenopodium album*	25 °C, 48 h, dry at 45 °C for 12 h	Not specified	TPC: V1 +73.7% (3.73 → 6.48 mg GAE/g), V2 +134.4% (2.41 → 5.65 mg GAE/g); AOA: V1 +26.5% (16.07 → 20.33%), V2 +29.1% (14.10 → 18.20%)	[13]
White fonio (*Digitaria exilis*), brown fonio (*Digitaria iburua*)	28 °C, 72 h, 92% RH, darkness, 7 h soaking	Not specified	TPC: brown +297.28% (0.19 → 0.76 mg GAE/g), white +279.27% (0.18 → 0.70 mg GAE/g); AOA DPPH: brown +78.24% (53.6 → 95.6 μmol TE/g), white +78.42% (51.9 → 92.5 μmol TE/g); AOA ORAC: brown +18.97% (31.1 → 37.0 μmol TE/g), white +20.10% (30.6 → 36.8 μmol TE/g)	[21]
Blue corn	26.9 °C, 207.7 h	Dry basis	TPC: +79% (1.88 → 3.36 mg GAE/g); ANTH: +9.9% (0.26 → 0.29 mg CGE/g); AOA ABTS: +192% (73.1 → 213.2 μmol TE/g); AOA ORAC: +160% (165.6 → 429.8 μmol TE/g); AOA DPPH: +148% (17.3 → 42.9 μmol TE/g)	[92]
Millet, amaranth, quinoa, other cereals	19–23 °C, 72 h, 92% RH, darkness, overnight soaking	Not specified	Polyunsaturated fatty acids: millet +1.6% (75.65 → 76.87%), amaranth +5.9% (59.13 → 62.59%), buckwheat +11.7% (46.92 → 52.43%); LINA: millet +0.5% (73.98 → 74.33%), amaranth +4.9% (56.70 → 59.50%); Omega-3: millet +67.2% (1.31 → 2.19%), amaranth +181.0% (0.89 → 2.50%), buckwheat +40.7% (2.68 → 3.77%)	[7]
Barnyard millet (*Echinochloa frumentacea* Link), foxtail millet, proso millet (*Panicum miliaceum* L.)	25 ± 2 °C, 48 h, 16 h soaking (1:3 *w*/*v*)	Not specified	TPC: proso +220.3% (0.74 → 2.38 mg FAE/g); TFC: foxtail +80.0% (0.88 → 1.58 mg CE/g); FERA: proso +67.1% (0.17 → 0.28 mg/g); AOA: foxtail +41.8% (117.3 → 68.3 μg/mL IC_50_); *α*-glucosidase inhibition: barnyard +59.1% (18.6 → 7.5 μg/mL IC_50_)	[93]
Kodo millet	25 °C, 48 h, 80–90% RH, 4 h soaking at 23–24 °C	Not specified	TPC: +52.2% (0.55 → 0.83 mg GAE/g); AOA: +48.3% (45.3 → 67.2 mg AAE/g); GABA: +410.6% (0.094 → 0.48 mg/g); AOA DPPH: +13.4% (67.3 → 76.3%); AOA H_2_O_2_: +69.7% (40.5 → 68.7 mmol TE/g)	[77]
Kodo millet and little millet	40 °C, 72 h, 80–90% RH, drying at 45 °C to 10% moisture	Dry basis	TPC: kodo +30% (values not specified), little +20%; TFC: kodo +50%, little +70%; AOA: kodo 88.46%, little 89.06%	[22]
Little millet	30 °C, 72 h, 90% RH, microwave drying 1050 W at 50 °C for 720 s	Not specified	TPC: +23.2% (2.95 → 3.64 mg GAE/g); TFC: +6.5% (2.02 → 2.15 mg CE/g); AOA: +35.45% (16.67 → 22.58%)	[116]
Pearl millet, finger millet, buckwheat	22 °C (buckwheat), 30 °C (millets), 72 h	Dry basis	TPC: finger −52.0% (0.29 → 0.14 mg GAE/g), pearl −42.0% (0.27 → 0.16 mg GAE/g), buckwheat +55.3% (0.28 → 0.43 mg GAE/g); AOA: buckwheat 22 °C +89.5% (28.2 → 53.5%), 30 °C +97.9% (17.3 → 34.3%); TAN: finger −82.5% (0.81 → 0.14 mg TAE/g), pearl +373.1% (0.40 → 1.87 mg TAE/g), buckwheat −33.4% (0.074 → 0.049 mg TAE/g)	[34]
Coix	29 °C, 24 h germination, 36 °C 10 h soaking	Not specified	GABA: +683% (0.027 → 0.21 mg/g); SOLP: +31.9% (16.0 → 21.2 mg/g); FAA: +41.3% (4.92 → 6.95 mg/g)	[67]
Seven grains: various cereals and buckwheat	16.5 °C, 98% RH, 120 h darkness, intermittent watering, aeration	Not specified	GABA: rye +700% (0.001 → 0.008 mg/mL); ARBX: wheat +33% (0.45 → 0.60 g/100g); *α*-amylase inhibition: barley +650% (3 → 35%); *α*-glucosidase inhibition: sorghum +25% (16 → 20%); AOA: rye +51% (spectrophotometric evaluation)	[27]
Wheat and triticale	24 °C germination, 1 mM GABA 3 h soaking	Dry basis	TPC: wheat +29% (1.62 → 2.09 μmol/g); ANTH: triticale +92% (0.38 → 0.73 units/g); Germination: +18–21%	[75]
Wheat, barley, sorghum	20 °C, 96 h	Dry basis	TPC: wheat +181% (0.36 → 1.01 μg GAE/mL), breakfast wheat +181% (0.72 → 2.02 μg GAE/mL), barley +69% (0.86 → 1.45 μg GAE/mL), breakfast barley +72% (1.38 → 2.38 μg GAE/mL), sorghum +102% (0.44 → 0.89 μg GAE/mL); AOA: wheat +107% (14.8 → 30.6% DPPH inhibition), barley +42% (19.2 → 27.4%), breakfast barley +158% (10.5 → 27.2%), sorghum +16% (15.7 → 18.2%)	[53]

Note. This table summarizes the effect of controlled germination as a physical inductor in various cereals and pseudocereals, detailing the optimal processing parameters and changes in bioactive compounds. The quantitative results show percentage changes compared to ungerminated grains. Abbreviations: AAE, Ascorbic acid equivalents; ABTS, 2,2’-azino-bis(3-ethylbenzothiazoline-6-sulfonic acid); AChE, Acetylcholinesterase; *α*-amylase, Alpha-amylase; *α*-glucosidase, Alpha-glucosidase; ANTH, Anthocyanins; AOA, Antioxidant activity; ARBX, Arabinoxylans; AVEN, Avenanthramides; AVN, Avenanthramides; *β*-GLU, *β*-Glucan; CAE, Caffeic acid equivalents; CATCH, Catechin; CE, Catechin equivalents; CGE, Cyanidin-3-glucoside equivalents; DPPH, 2,2-diphenyl-1-picrylhydrazyl; FAA, Free amino acids; FAE, Ferulic acid equivalents; FERA, Ferulic acid; FeSO_4_, Iron sulfate; FPHE, Free phenolic compounds; FRAP, Ferric reducing antioxidant power; GABA, *γ*-Aminobutyric acid; GACA, Gallic acid; GAE, Gallic acid equivalents; H_2_O_2_, Hydrogen peroxide; HESP, Hesperidin; IC_50_, Half maximal inhibitory concentration; LINA, Linoleic acid; OLEA, Oleic acid; ORAC, Oxygen radical absorbance capacity; *p*-COU, *p*-Coumaric acid; *p*-HBA, *p*-Hydroxybenzoic acid; PHEA, Phenolic acids; POLY, Polyphenols; QE, Quercetin equivalents; RE, Rutin equivalents; RUTI, Rutin; SOLP, Soluble proteins; TAE, Tannic acid equivalents; TAN, Tannins; TE, Trolox equivalents; TFC, Total flavonoid content; TPC, Total phenolic content; VITX, Vitexin.

**Table 4:** Physical inducers for germination of cereals and pseudocereals III: electromagnetic and pressure technologies for the accumulation of bioactive compounds.

Type of Inductor	Species Studied	Optimal Parameters	Measurement Basis	Bioactive Compounds and Quantitative Results	Ref.
PAW	Wheat	PAW-3 treatment, 15 mm distance, Ar-O_2_ gas (98% Ar, 2% O_2_), 40 L/min, 600 W	Not specified	TPC: +10.46% (values not specified); CHL-*a*: +89.46% (values not specified); CHL-*b*: +112.46% (values not specified); CAROT: +91.58% (values not specified); SOLP: +19.48% (values not specified); GABA: +32.56% (values not specified); FAA: +28.23% (values not specified); SOD: +47.12% (values not specified)	[37]
PAW with APPJ	Barley	Treatment C: 30 min APPJ, 4.5 h soaking, 19 h air rest, 15 °C, 76% RH	Dry basis	*β*-AMY: +18.8% (values not specified); Germination: improved acrospire growth	[45]
HHP and soaking	Buckwheat	Soaking 40 °C 4 h, 600 MPa 30 min single cycle	Dry basis	TPC: +16% (values not specified); AOA: +2.5% (values not specified)	[98]
CHVEF, AHVEF, PHVEF	Winter triticale	AHVEF (3 kV·cm^−1^, 60 s) for germination energy and uniformity	Not specified	Root length: +28.7% (9.4 → 12.1 cm); Grains per spike: +31.0% (values not specified); Grain yield: +57.8% (values not specified); Germination uniformity: +4% (94.7 → 98.7%)	[121]
PEF	Wheat	PEF 6 kV·cm^−1^, 50 pulses prior to imbibition	Wet basis	TPC: +18.56% (2.80 → 3.32 mg GAE/L); CHL: +373% (2.8 → 10.8 mg/g); CAROT: +34% (2.1 → 2.8 mg/g); SOLP: +12.08% (8.94 → 10.02 mg/g); AOA: +5.78% (1314.4 → 1390.3 μmol TE/L)	[6]
PEF	Barley	10 min pre-soaking in phosphate buffer, PEF 3.8 kV·cm^−1^, 100 pulses, 20 *μ*s width	Dry basis/Wet basis	*α*-AMY: −4% (211 → 203 U/g); *β*-GLUC: +12% (448 → 503 U/kg); Diastatic power: +2% (251 → 255 WK units) with optimized treatment; *α*-AMY: −73% (240 → 66 U/g); *β*-GLUC: −87% (603 → 80 U/kg); Diastatic power: −45% (364 → 202 WK units) with non-optimized treatment	[107]
PEF	Wheat	PEF 3 kV·cm^−1^, 200 pulses (19.8 kJ/kg) before first hydration cycle, or 100 pulses (9.9 kJ/kg) after first cycle	Wet basis	*α*-AMY: +104% (34.4 → 70.6 U/g); *β*-AMY: +25% (15.2 → 19.1 U/g); Water absorption: +25% (hydration rate); Water retention: +15% (values not specified)	[44]
PEF	Wheat	161.8 Hz, 6.1 J, 19.5 s	Not specified	Germination rate: +10% (values not specified); Normal seedlings: +28% (72 → 100%)	[43]
Static magnetic field	Triticale	3.72 mT, 6 h, 10 °C	Not specified	Maximum germination: +9% (87 → 96%); Time to 50% germination: −12.4% (25.9 → 22.7 h); Time to 75% germination: −16% (33.2 → 27.9 h)	[122]
Static magnetic field	Brown rice	10 mT, 60 min, 25 °C, followed by 24 h germination at 30 °C in 5–10 mM GABA	Dry basis	GABA: +207.6% (16.43 → 50.54 mg/100g); GABA-T activity: −16.14% (63 → 53 μg/g); Root length maintained <3 mm (2.03–2.45 mm)	[15]
HPCD	Barley	57 bar, 25–35 °C	Wet basis	Oat germination: −13.8% (58 → 50%) to −100% (58 → 0%); Barley germination: −100% (11 → 0%)	[120]
Gamma irradiation	Various millet varieties	2.5 kGy, 12% moisture	Dry basis	TPC: +24.5% (16.85 → 21.04 mg GAE/g); DPPH: +55.6% (48.91 → 76.10% inhibition); Reducing power: +120.8% (0.24 → 0.53 μmol AAE/g)	[111]
Microwave irradiation	Tartary buckwheat	300 W, 50 s, 25 °C, 85% RH, 5 days darkness	Dry basis	FLVN: +31.78% (3.91 → 5.15 g/100g); PAL: +6.50% (values not specified); CHI: +8.64% (values not specified); FLS: +14.55% (values not specified); AOA: significant increase	[104]
Microwave	Bitter buckwheat	600 W, 10 s before 7 days germination, 25 ± 2 °C, 85% RH	Not specified	TFC: +377% (11.3 → 53.9 mg CE/g), AOA DPPH: +264% (17 → 62%), FAA: +427% (2.13 → 11.24 mg/g), CAT: +300% (7.7 → 30.7 mg H_2_O_2_/g FW min), SOD: +58% (11.4 → 18.0 U/g)	[71]
Ultra-high frequency microwave (UHF EMF)	Spring barley	0.42 kW, 11 s, 7 days at 18 °C, 60% RH	Not specified	CAFA in leaves: +95.2% (values not specified); FERA in leaves: +50.7% (values not specified); VANA in leaves: +329.3% (values not specified); SYRA in endosperm: +1871% (values not specified); TPC in leaves: +167.6% (values not specified)	[106]
Light (different intensities)	Bitter buckwheat	20 °C, 99% RH, 6000 lux for rutin/flavonoids, 600 g buckwheat/plate, 5° inclination	Not specified	RUTI: +34% (883.87 → 1,184.33 mg/L); MYR highest at 6000 lux (37.37 mg/L); QUER highest at 6000 lux (62.73 mg/L); KAEM: +12% (16.85 → 18.87 mg/L); TPC: +47% (824.61 → 1,213.04 mg/L); TFC: +64% (838.82 → 1,379.79 mg/L); AOA DPPH: +361% (11.06 → 50.96%); AOA ABTS: +250% (8.77 → 30.69%)	[47]
PL	Corn	6 h soaking, 400 pulses (0.50 J/cm^2^), 30 °C, 90% RH, 48–72 h	Dry basis	GABA: +27.20% (31.73 → 40.36 mg/100g); GABA vs. non-germinated: +801% (values not specified); GLUAS: +11.79% (39.96 → 44.67 mg/100g); FAA: +239.65% (83.55 → 283.78 mg/100g); GAD: significant increase; GABA-T: significant reduction	[9]
PLT	Brown rice	300 pulses at 400 J, 28 °C, 95% RH, 36 h	Not specified	GABA: >30% increase in all varieties (29.6–40.4 → 39–53 mg/100g); *OsbZIP56* gene: +20% GABA increase (33 → 41 mg/100g)	[68]
Temperature and photoperiod (light)	Common buckwheat	16 °C, 20/4 h light/dark photoperiod, 288 h	Not specified	TPC: +76.6% (0.96 → 1.70 mg GAE/g); TFC: +20% (4.16 → 5.00 mg QE/g); CAROT: +18.19% (0.38 → 0.45 mg/g) with 16 °C vs. 25 °C; CAROT: +21.34% (values not specified) with extended photoperiod; CHL: +35.40% (0.74 → 1.00 mg/g) with extended photoperiod; AOA: +15% (75 → 90% inhibition)	[36]

Note. This table summarizes the effect of different physical inducers based on electromagnetic fields and pressure treatments applied during the germination of cereals and pseudocereals to enhance the accumulation of bioactive compounds. Quantitative results show the percentage changes compared to untreated controls. Abbreviations: AAE, Ascorbic acid equivalents; ABTS, 2,2-diphenyl-1-picrylhydrazyl; AHVEF, Alternating High Voltage Electric Field; *α*-AMY, *α*-Amylase; AOA, Antioxidant activity; APPJ, Atmospheric pressure plasma jet; *β*-AMY, *β*-Amylase; *β*-GLUC, *β*-Glucanase; CAFA, Caffeic acid; CAT, Catalase; CAROT, Carotenoids; CE, Catechin equivalents; CHI, Chalcone isomerase; CHL, Chlorophyll; CHL-*a*, Chlorophyll *a*; CHL-*b*, Chlorophyll *b*; CHVEF, Constant High Voltage Electric Field; DPPH, 2,2-diphenyl-1-picrylhydrazyl; EMF, Electromagnetic field; FAA, Free amino acids; FERA, Ferulic acid; FLS, Flavonol synthase; FLVN, Flavonoids; GABA, *γ*-Aminobutyric acid; GABA-T, *γ*-Aminobutyric transaminase; GAD, Glutamate decarboxylase; GAE, Gallic acid equivalents; GLUAS, Glutamine synthetase; H_2_O_2_, Hydrogen peroxide; HHP, High hydrostatic pressure; HPCD, High-pressure carbon dioxide; KAEM, Kaempferol; MYR, Myricetin; *OsbZIP56*, Transcription factor; PAL, Phenylalanine ammonia-lyase; PAW, Plasma-activated water; PEF, Pulsed electric field; PHVEF, Pulsed High Voltage Electric Field; PL, Pulsed light; PLT, Pulsed light treatment; QUER, Quercetin; RUTI, Rutin; SOD, Superoxide dismutase; SOLP, Soluble proteins; SYRA, Syringic acid; TE, Trolox equivalents; TFC, Total flavonoid content; TPC, Total phenolic content; UHF, Ultra-high frequency; VANA, Vanillic acid; WK, Windisch-Kolbach units.

**Table 5:** Physical inducers for germination of cereals and pseudocereals IV: radiation, plasma, and ultrasound technologies for the accumulation of bioactive compounds.

Type of Inductor	Species Studied	Optimal Parameters	Measurement Basis	Bioactive Compounds and Quantitative Results	Ref.
UV-A LED Light	Sorghum	35 °C, 98% RH, 28 h germination, 36 h sprouting, 11.9 h UV-A at 5.1 μW·cm^−2^	Wet basis	TPC: +143.57% (0.67 → 2.08 mg GAE/g); FPHE: +210.45% (22.4 → 48.8%); AOA: +168.86% (5.62 → 15.11 μmol TE/g)	[49]
UV-B light (280–311 nm)	Mexican blue corn	26.9 °C, 80–90% RH, 207.7 h, UV-B 37.0 h after 96 h	Dry basis	TPC: +587.2% (2.42 → 16.62 mg GAE/g); FPHE: +1148% (0.42 → 5.25 mg GAE/g); BPHE: +469% (2.00 → 11.37 mg GAE/g); ANTH: +29.9% (0.30 → 0.36 mg C3GE/g); GABA: +199.9% (0.098 → 0.294 mg/g); AOA ABTS: +133.9% (12.42 → 29.06 mmol TE/100g); AOA DPPH: +173.4% (4.09 → 11.17 mmol TE/100g)	[26]
UV-B light (wavelengths between 280 and 315 nanometers)	Highland barley	Germination: 72 h at 25 °C. UV-B radiation: 0.2 W m^−2^ for 6 h/day for flavonoids; 0.2 W m^−2^ for 6 h/day for polyphenols; 0.2 W m^−2^ for 12 h/day for riboflavin; 0.2 W m^−2^ for 3 h/day for GABA.	Dry basis	TPC: +49.4% (values not specified); GABA: +40.21% (values not specified); RIBO: reached 2.5 μg/g after 72 h; *β*-GLU: −20.15% (values not specified)	[73]
UV-B	Buckwheat or common buckwheat	28.7 °C, 3.0 days, UV-B 30.0 *μ*mol·m^−2^·s^−1^ for 7.6 h/day	Not specified	FLVN: +97% (0.95 → 1.87 mg/g); TPC: +54% (12.5 → 19.3 μg GAE/g); AOA DPPH: +54% (25 → 53% scavenging); AOA ABTS: +66% (42 → 80% scavenging); AOA FRAP: +54% (30 → 48% scavenging)	[74]
UV-C light (200–280 nm)	Amaranth	3 cm distance, 15 min exposure	Dry basis	TPC: +196% (0.45 → 1.33 mg GAE/g); *p*-CQA: +17.7% (893.4 → 1058.8 area units); TRYP: +12.4% (7977.5 → 8969.5 area units)	[80]
UV-C light (254 nm)	Wheat and Chia	UV-C 120 min chia (35.7 × 10^4^ J m−^2^), 180 min wheat (141.7 × 10^4^ J m^−2^), germination 25 °C	Wet basis	AOA chia: +317% (1.8 → 7.5 g TE/kg); AOA wheat: +78% (0.9 → 1.6 g TE/kg); TPC: no significant effect	[28]
Plasma (low-pressure plasma and sliding arc plasma)	Barley, Wheat, Triticale	Low-pressure plasma, 5 min	Dry basis	Low-pressure plasma: +18% germination; Atmospheric plasma: −58% germination	[110]
Cold Atmospheric Pressure Plasma (CAPP)	Barley	10–20 s for ambient air/nitrogen, 10–30 s for oxygen, 24 ± 2 °C	Wet basis	SOD: +40% (values not specified); G-POX: +132% (values not specified); Germination acceleration: +56%; Root growth: +20.6%; Sprout weight: +19%	[88]
Dielectric Barrier Discharge (DBD) air atmospheric plasma	Rice	DBD plasma for 60 s	Not specified	Germination: +9.0% (85 → 92.7%); Vigor index: +18.0% (510 → 602); Germination speed: +7.3% (12.4 → 13.3); CHL-*b*: +10.3% (0.146 → 0.161 mg/g); CAROT: +7.6% (0.144 → 0.155 mg/g)	[123]
Surface Barrier Discharge (SBD) plasma	Various winter and spring cereals	SBD plasma for 60 min (winter wheat), 24 °C	Not specified	Shoot length wheat: +31% (16 → 21 mm); Root length wheat: +15% (104 → 120 mm, 30 min), +33% (104 → 138 mm, 60 min), +21% in 6 days (310 → 375 mm)	[46]
Atmospheric pressure plasma (SDBD)	Barley	6 min SDBD plasma, 51.7 W, 8 kV*p*-p, 14.4 kHz, 15 °C, 16/8 h light/dark	Not specified	TPC: +9% (2.15 → 2.35 mg/g); SAPO: +50% (0.60 → 0.90 mg/g); GABA: +40% (0.11 → 0.15 mg/g); POLI: +90% (0.42 → 0.80 mg/g)	[70]
Cold plasma (CP) and electromagnetic field (EMF)	Common buckwheat	CP7 for ‘VB Nojai’, CP5 for ‘VB Nojai’, EMF15 for ‘VB Vokiai’	Not specified	In vitro germination: 100% in all groups; Germination time: −7% (43.6 → 40.6 h); Field emergence: −13% to −20%	[103]
Cold plasma (DBD)	Brown rice	Plasma at 135 W, 75 s, argon flow 22 mL/min, germination 25–28 °C, 1–1.5 days	Wet basis	TPC: +86% (0.786 → 1.461 mg GAE/g); TOCO: +290% (11.50 → 44.85 μg/g); *γ*-ORY: +80% (40.91 → 73.64 μg/g); ANTH: +38% (212.26 → 292.76 μg/g); PHYS: +40.6% (4.04 → 5.68 mg STE/100g); TERP: +80.5% (4.42 → 7.98 mg STE/100g)	[90]
Cold plasma (microwave discharges)	Barley, corn	Barley: Ar-20%O_2_ 180 s at 4 mbar; corn: Ar-20%O_2_ 240 s + N_2_-2%O_2_ 120 s at 8 mbar	Not specified	No significant effect on germination; Slight positive effect on root/shoot length	[109]
Atmospheric cold plasma (CAP): DBD and APPJ	Various cereals	APPJ Ar + O_2_ 11 days for barley, APPJ Ar + air 10 days for wheat	Dry basis	Root dry mass rye: +15.6%; Barley: +16.2%; Wheat: +14.3%; Germination rate barley: +21.4%; Oats: +28.8%; Wheat: +33.3%	[105]
US	Oat	Soaking 4 h at 23–24 °C, ultrasound 5 min at 25 kHz (16 W/L), germination 72–96 h at 24 ± 2 °C, 95 ± 3% RH	Dry basis	GABA: +256.9% (0.632 → 2.253 mg/g); AVEN 2c: +3403.2% (6.43 → 225.27 μg/g); AVEN 2p: +2024.6% (5.53 → 117.49 μg/g); AVEN 2f: +1267.6% (5.11 → 69.85 μg/g); TPC: +11.24% (14.93 → 16.61 μg GAE/mg); AOA: +72.45% (39.34 → 67.84% DPPH)	[11]
US	Corn	45 kHz, 30 °C, 30 min, germination at 30 °C, 90% RH for 60 h	Wet basis	GABA: +30.55% (0.311 → 0.406 mg/g)	[17]
US (40 kHz, 30 min)	Brown rice	40 kHz, 30 min, germination 36 h at 28 ± 1 °C	Not specified	Germination: +28% (77 → 100%); Metabolomic profile changes (specific values not reported)	[108]
High intensity ultrasound (HIU)	Brown rice	HIU: 28 kHz, 17.83 W/cm^2^, 5 min, germination at 37 °C for 32 h	Dry basis	GABA: +56.92% (114.68 → 179.85 mg/kg); Amino acid index: +137.5% (0.8 → 1.9%); AOA FRAP: +43.9% (4.13 → 5.94 μmol Fe^2+^/g); Iron bioaccessibility: +147.1% (26.6 → 38.6%)	[31]
US and controlled germination	Red spring wheat, white soft wheat	28 ± 2 °C, 95 ± 3% RH, 6 h soaking, 72 h germination, 30 min ultrasound at 25 kHz	Wet basis	GABA: +339% (14.68 → 49.72 mg/100g); GABA with ultrasound: +30.7% (49.72 → 64.98 mg/100g); SOLDF in SW: −18.4% (2.17 → 1.77 g/100g); Glucose in HW: +471% (74.21 → 423.58 mg/100g)	[10]

Note. This table summarizes the effect of different physical inducers based on radiation, plasma and ultrasound applied during the germination of cereals and pseudocereals to enhance the accumulation of bioactive compounds. The quantitative results show percentage changes compared to untreated controls. Abbreviations: ABTS, 2,2’-azino-bis(3-ethylbenzothiazoline-6-sulfonic acid); ANTH, Anthocyanins; AOA, Antioxidant activity; APPJ, Atmospheric pressure plasma jet; AVEN, Avenanthramides; *β*-GLU, *β*-Glucan; BPHE, Bound phenolic compounds; C3GE, Cyanidin-3-glucoside equivalents; CAP, Cold Atmospheric Plasma; CAPP, Cold Atmospheric Pressure Plasma; CAROT, Carotenoids; CHL-*b*, Chlorophyll *b*; CP, Cold plasma; DBD, Dielectric Barrier Discharge; DPPH, 2,2-diphenyl-1-picrylhydrazyl; EMF, Electromagnetic field; FLVN, Flavonoids; FPHE, Free phenolic compounds; FRAP, Ferric reducing antioxidant power; GABA, *γ*-Aminobutyric acid; GAE, Gallic acid equivalents; G-POX, Guaiacol peroxidase; HIU, High intensity ultrasound; HW, Hard wheat; *p*-CQA, *p*-Coumaroylquinic acid; PHYS, Phytosterols; POLI, Policosanols; RIBO, Riboflavin (Vitamin B2); SAPO, Saponarin; SBD, Surface Barrier Discharge; SDBD, Surface Dielectric Barrier Discharge; SOD, Superoxide dismutase; SOLDF, Soluble dietary fiber; STE, Sitosterol equivalents; SW, Soft wheat; TE, Trolox equivalents; TERP, Terpenes; TOCO, Tocopherols (Vitamin E); TPC, Total phenolic content; TRYP, Tryptophan; US, Ultrasound; UV-A, Ultraviolet-A radiation; UV-B, Ultraviolet-B radiation; UV-C, Ultraviolet-C radiation; *γ*-ORY, *γ*-Oryzanols.

**Table 6:** Chemical and biochemical inducers for the germination of cereals and pseudocereals: elicitors for the maximization of bioactive compounds.

Type of Inducer	Species Studied	Optimal Processing Parameters	Measurement Basis	Bioactive Compounds and Quantitative Results	**Ref.**
Vegetable salts (ashes)	Corn	At*p*-Y: 25.12 h at 25.54 °C, 0.52% salt, 144.37 h germination, 37.65 h maturation. Coca-sr: 1.608 h at 36.63 °C, 1.11% salt, 144.37 h germination, 27.07 h maturation	Dry basis	TPC Coca-sr: +72.4% (48.77 → 84.08 mg GAE/g); TFC Coca-sr: +126.4% (39.55 → 89.53 mg CE/g); AOA DPPH Coca-sr: +89.9% (12.13 → 23.04%); AOA FRAP Coca-sr: +193.6% (12.16 → 35.70 μmol TE/g)	[2]
Chitosan, jasmonic acid (JA), SA	Common buckwheat	Chitosan 0.1%, JA 150 *μ*M, 72 h, 25 °C	Not specified	Chitosan: TPC: +23% (0.74 → 0.91 mg GAE/g); GACA: +51% (6.09 → 9.19 μg/g); CATCH: +72% (56.18 → 96.59 μg/g); CHLA: +69% (58.92 → 99.66 μg/g); EPIC: +122% (44.44 → 98.51 μg/g); JA: TPC: +147% (0.74 → 1.82 mg GAE/g); RUTI: +138% (424.42 → 1011.3 μg/g); CAFA: +48% (77.99 → 115.63 μg/g); EPIC: +695% (44.44 → 353.28 μg/g)	[112]
Slightly acidic electrolyzed water (SAEW)	Brown rice	SAEW pH 5.5–6.0, redox potential 940–968 mV, available chlorine 10 ppm, 35 ± 1 °C, 85% RH, 48 h darkness	Dry basis	TPC: +743% (13.75 → 115.94 mg GAE/100g); TFC: +578% (14.72 → 99.85 mg CE/100g); GABA: +299% (1.84 → 7.35 mg/L); FERA: +2751% (4.46 → 127.2 μg/g); *p*-COU: +1339% (3.74 → 53.8 μg/g); ASCA: detected only in SAEW (224.4 μg/g); QUER (SAEW): −69% (2.82 → 0.86 μg/g); QUER (water): +154% (2.82 → 7.17 μg/g); AOA DPPH: +839% (15.53 → 145.99 mg TE/100g); AOA ABTS: +792% (17.06 → 152.21 mg TE/100g); AOA FRAP: +934% (16.11 → 166.61 mg TE/100g)	[25]
Hydrogen-rich water (HRW)	Wheat	HRW 4 h, 23 ± 2 °C, 50% RH	Not specified	Germination: +21.1% (79.99 → 96.66%); Vigor index: +84.7% (146 → 269.7); Chlorophyll: +76.8% (6.6 → 11.69 mg/g)	[113]
3% sucrose and 7.5 mM calcium chloride	Common buckwheat	3% sucrose + 7.5 mM CaCl_2_, sprayed every 6 h, 8 days, 25 °C, 60% RH, darkness	Wet basis	TPC: +64% (2.99 → 4.90 mg GAE/g); TFC: +58% (1.16 → 1.83 mg CE/g); GABA: +59% (37.6 → 59.7 mg/kg); ORI: +43% (175.5 → 251.3 mg/kg); ISOV: +30% (331.4 → 431.5 mg/kg); VITX: +36% (279.9 → 380.6 mg/kg); ISOV: +38% (437.4 → 603.3 mg/kg); RUTI: +34% (288.9 → 387.0 mg/kg); ASCA: +10% (130.5 → 143.2 mg/kg); *α*-TOCO: +31% (1.3 → 1.7 mg/kg); AOA DPPH: +51% (3.21 → 4.85 mg TE/g)	[33]
Acidic medium	Brown rice	pH 2.7, 25 °C, 12 h	Not specified	PHYA: −46.2% (5.54 → 2.98 g/kg); PHYT: +402% (112.36 → 563.89 U/kg); Calcium bioaccessibility: +32.9% (18.84 → 25.04%); Zinc bioaccessibility: +44.4% (19.56 → 28.24%)	[38]
Gaseous ozone (O_3_)	Spring malting barley	50 ppm, 1–6 h, 20 °C, 98% RH, 19.8% grain moisture	Dry basis	Germination energy: 96.0% vs. 99.3% in control; Significant reductions after 1 h	[124]
Gaseous ozone (O_3_)	Wheat	Ozone: 3 h at 50 ppm, flow 1 L min^−1^; Soaking: 24 h at 22 ± 2 °C (1:2); Germination: 72 h at 22 ± 2 °C, 80% RH, darkness	Dry basis	TPC grains: +1.5% (3 h), −56.2% (4 h), −54.8% (5 h); TPC germinated: −15.5% (3 h), −19.5% (4 h), −18.9% (5 h); AOA grains: +2.4% (3 h), −13.0% (4 h), −10.0% (5 h)	[19]
Citric and lactic acid	Adlay	1% citric acid, 12 h at 25 °C, germination 60 h at 25 °C, 95% RH, darkness	Dry basis	TPC citric acid: +18.3% (2.36 → 2.79 mg GAE/g); TPC lactic acid: −14.9% (2.36 → 2.01 mg GAE/g); TFC citric acid: +17.0% (0.55 → 0.65 mg RE/g); TFC lactic acid: −9.0% (0.55 → 0.50 mg RE/g); AOA citric acid: +39.1% (118.44 → 164.74 μmol TE/g); AOA lactic acid: −16.8% (118.44 → 98.55 μmol TE/g)	[114]
Sodium Chloride (NaCl)	Wheat	48 h at 17 °C, 80% RH, darkness, 60 mM NaCl solution	Dry basis	TPC: +242.3% (1.37 → 4.70 mg GAE/g)	[72]
Sodium Chloride (NaCl) and CaCl_2_ supplement	Yellow corn	16 h water soaking, 8 h 300 mM NaCl + 5 mM CaCl_2_, 72 h germination at 24 ± 1 °C in darkness	Dry basis	LUT NaCl+CaCl_2_: +21.50% (9.44 → 11.47 μg/g); LUT CaCl_2_: +36.55% (9.44 → 12.89 μg/g); ZEAX NaCl+CaCl_2_: +30.18% (6.23 → 8.11 μg/g); *α*-CRY NaCl + CaCl_2_: +23.33% (1.50 → 1.85 μg/g); AOA ORAC NaCl: +127.80% (36.54 → 83.24 μmol TE/g)	[87]
Sodium chloride (NaCl)	Quinoa	300 mM NaCl, 24 °C, 240 h (10 days)	Wet basis	TPC sprouts: +152% (1.02 → 2.57 mg GAE/g); TFC sprouts: +243% (0.23 → 0.79 mg CE/g); FLVL sprouts: +92% (0.26 → 0.50 mg QE/g); ANTH sprouts: +238% (2.47 → 8.36 μg CGE/g); AOA DPPH sprouts: +3700% (values not specified)	[89]
Sodium silicate and iron chelate (Fe-EDTA)	Common buckwheat	7 days, 4 mM sodium silicate (SIL) or SIL + 0.5 mM Fe-EDTA (SIL-Fe), 15 min immersion twice daily	Dry basis	FLVN SIL-Fe: −20.6% (5.094 → 4.044 mg/g); PHEA SIL: +11.2% (6.114 → 6.799 mg/g); EPIC SIL-Fe: −24.2% (3.529 → 2.674 mg/g); CAFA esters SIL: +80.8% (0.915 → 1.654 mg/g); Fe SIL-Fe: +335% (81.12 → 353.0 μg/g); Si SIL-Fe: +204% (152.6 → 464.2 μg/g)	[91]
Zinc oxide nanoparticles (ZnO NPs)	Pearl millet	150 ppm ZnO NPs, 6 h imbibition, 28 °C	Not specified	Germination: +20% (73.2 → 93.4%); Vigor index: +51% (1284.6 → 1944.5)	[125]
Zinc oxide nanoparticles (ZnONPs)	Corn	700–1000 mg/L ZnONPs for germination, 800 mg/L for CAROTS	Not specified	CHL: +170.8% (0.252 → 0.683 mg/g); TPC: +3.4% (values not specified); CAROT: +221.5% (values not specified); PROL: +66.8% (0.206 → 0.344 mg/g)	[97]
Gibberellic acid (GA_3_) and abscisic acid (ABA)	Wheat	GA_3_ 150 *μ*M, 12 h, 25 °C, 16 h photoperiod	Not specified	*α*-AMY Yangmai 13: +13.5 mg g^−1^ min^−1^; *α*-AMY Yannong 19: +12.5 mg g^−1^ min^−1^	[40]
Phytohormones (IAA, SA, GA)	Wheat	IAA: 0.01 mg/mL, GA: 0.01 mg/mL, SA: 0.001 mg/mL or combination, 72–120 h, room temperature, darkness	Not specified	TPC IAA+GA+SA: +128% (values not specified); TFC IAA+GA+SA: +182% (values not specified); FERA: +949.3% (537,589 → 5.1 × 10^8^ area units); NARG: +438.3% (102,023 → 4 × 10^7^ area units); TRIC: +76% (191,663 → 1.5 × 10^7^ area units); GABA: −64% (2 × 10^10^ → 6 × 10^9^ area units); AOA DPPH: +106% (values not specified); AOA FRAP: +108% (values not specified)	[39]
Vitamin B6 (Pyridoxal phosphate, PLP)	Bitter buckwheat	2 days at 22 °C, 75% RH, darkness, then 2.5 mM PLP at 30 °C for 24 h	Not specified	GABA: +867% (0.39 → 3.82 g/kg); GLUAS: +175% (1.91 → 5.26 g/kg); FLVN: +11% (14.93 → 16.59 g/kg); TPC: +33% (17.33 → 23.09 g/kg); AOA DPPH: +23% (67.54 → 82.81%); AOA ABTS: +31% (38.75 → 50.83%); ACE inhibition: +135% (32.86 → 77.26%)	[30]

Note. This table summarizes the main chemical inducers used to enhance bioactive compounds during the germination of cereals and pseudocereals, detailing the optimal processing conditions, mechanisms of action, and quantitative results. Percentage values indicate the increase or decrease compared to the control. Abbreviations: ABA, Abscisic acid; ABTS, 2,2′-azino-bis(3-ethylbenzothiazoline-6-sulfonic acid); ACE, Angiotensin-converting enzyme; ANTH, Anthocyanins; *α*-AMY, *α*-Amylase; AOA, Antioxidant activity; ASCA, Ascorbic acid; CAFA, Cafeic acid; CAROT, Carotenoids; CAROTS, Carotenoids; CATCH, Catechin; CE, Catechin equivalents; CGE, Cyanidin-3-glucoside equivalents; CHL, Chlorophyll; CHLA, Chlorogenic acid; CaCl_2_, Calcium chloride; DPPH, 2,2-diphenyl-1-picrylhydrazyl; EPIC, (-)-Epicatechin; FERA, Ferulic acid; FLVL, Flavonols; FLVN, Flavonoids; FRAP, Ferric reducing antioxidant power; Fe, Iron; GA3, Gibberellic acid; GABA, *γ*-Aminobutyric acid; GACA, Gallic acid; GAE, Gallic acid equivalents; GLUAS, Glutamine synthetase; HRW, Hydrogen-rich water; IAA, Indoleacetic acid; ISOV, Isovitexin; JA, Jasmonic acid; LUT, Lutein; NARG, N-Acetylglutamine; NPs, Nanoparticles; NaCl, Sodium chloride; O_3_, Ozone; ORAC, Oxygen radical absorbance capacity; ORI, Orientin; PHEA, Phenolic acids; PHYA, Phytic acid; PHYT, Phytases; PLP, Pyridoxal phosphate; PROL, Proline; QE, Quercetin equivalents; QUER, Quercetin; RE, Rutin equivalents; RH, Relative humidity; RUTI, Rutin; SA, Salicylic acid; SAEW, Slightly acidic electrolyzed water; SIL, Silicate; Si, Silicon; TE, Trolox equivalents; TFC, Total flavonoid content; TPC, Total phenolic content; TRIC, Trichloroacetic acid; VITX, Vitexin; ZEAX, Zeaxanthin; ZnO, Zinc oxide; ZnONPs, Zinc oxide nanoparticles; *α*-CRY, *α*-Cryptoxanthin; *α*-TOCO, *α*-Tocopherol; *p*-COU, *p*-Coumaric acid.

**Table 7:** Biological inducers for the germination of cereals and pseudocereals: microbial fermentation and derivatives for the maximization of bioactive compounds.

Type of inducer	Species Studied	Optimal Parameters	Measurement basis	Bioactive Compounds Analyzed and Quantitative Results	**Ref.**
Cyanobacteria extracts and biologically synthesized silver nanoparticles (AgNPs)	Barley and wheat	AgNPs synthesized with 1.0 mM AgNO_3_ at 30 °C in light, smallest particles (7.3–28 nm)	Not specified	GRI barley cv. Giza 123: +3.7% (18.6 → 19.3%); GVC barley cv. Giza 123: +1.1% (55.7 → 56.3%); MGT barley cv. Giza 123: −7.1% (3.0 → 2.7 days)	[81]
Fermentation, soaking and controlled germination	Corn	Soaking: 24 h at 18 °C (1:3 *w*/*v*), germination: 80 h at 18 °C, fermentation: 24 h at 30 °C with *L. plantarum* 299v	Dry basis	PHYA FL*p*-SG: −85.6% (9.58 → 1.39 g/kg); PHYA FYLc: −68.7% (9.58 → 3.02 g/kg); PHYA FLp: −65.3% (9.58 → 3.35 g/kg); PHYA FSp: −51.8% (9.58 → 4.65 g/kg); PHYA germination: −31.9% (9.58 → 6.35 g/kg); PHYA soaking: −12.6% (9.58 → 8.44 g/kg); Phytate molar ratio FL*p*-SG: −81% (40.76 → 7.77); Phytate molar ratio FL*p*-SG: −85% (41.42 → 6.24)	[4]
Spontaneous fermentation and germination	Red sorghum and pearl millet	Germination: 48 h at 30 °C with intermittent water spraying, drying at 50 °C for 12 h, fermentation: 48 h at 30 °C	Dry basis	Red sorghum (G+F): TPC: −69% (82.23 → 25.49 mg GAE/g); TFC: −54% (23.83 → 10.93 mg CE/g); TDC: −89.2% (9.06 → 0.98 mg/g); PHYA: −90.1% (2.10 → 0.21 g/kg); AOA DPPH: −30% (81.16 → 56.80%); AOA ABTS: +3% (89.99 → 92.71%); Pearl millet (G+F): TPC: −26.3% (19.15 → 14.11 mg GAE/g); TFC: −56.9% (8.85 → 3.81 mg CE/g); TDC: −86.1% (1.01 → 0.14 mg/g); PHYA: −85.1% (2.58 → 0.38 g/kg); AOA DPPH: −69.1% (69.28 → 21.38%); AOA ABTS: +11% (84.39 → 93.64%)	[24]
Fermentation with *Rhizopus oligosporus* (SSF) in two systems: traditional plate fermentation (PF) and bioreactor fermentation (BF)	djulis	Germination: 4 days at room temperature (42.6 ± 9.5 mm sprouts), fermentation: bioreactor at 35 °C, 0.4 vvm aeration, 5 rpm rotation, 4 days	Not specified	AOA: +101% (19.26 → 38.76 mM TE); FAA: +172% (36.62 → 99.56 mg casein tryptone/g); FPHE: +23% (BF vs. PF); CAROT: +37% (BF vs. PF); CHL-*a*: +13% (BF vs. PF); CHL-*b*: +133% (BF vs. PF); ANTH: +134% (BF vs. PF)	[41]
Fermentation and controlled germination	Naked barley	Germination: 30 °C, 48 h, 80–85% RH; germination-fermentation: 48 h germination + 24 h fermentation at 35 °C	Not specified	GABA germination: +116.63% (5.49 → 11.9 mg/100 g); GABA germination-fermentation: +87.53% (5.49 → 10.3 mg/100 g); GABA soaking: +78.51% (5.49 → 9.8 mg/100 g); TPC germination-fermentation: +68.39% (16.85 → 28.37 mg GAE/g); TPC germination: +26.21% (16.85 → 21.27 mg GAE/g); AOA DPPH germination-fermentation: +267.46% (values not specified); AOA DPPH germination: +146% (values not specified); AOA ABTS germination-fermentation: +36.1% (3.13 → 4.26 mg TE/g); *β*-GLU germination: −9.68% (5.99 → 5.41%); *β*-GLU germination-fermentation: −5.51% (5.99 → 5.66%)	[5]
Natural fermentation, soaking and roasting	Blue corn	Soaking 16 h, germinate at 25 °C for 72 h with periodic watering	Not specified	TPC germination: +36.02% (44.88 → 61.05 mg GAE/100 g); TPC fermentation: +14.88% (44.88 → 51.56 mg GAE/100g); TPC roasting: −20.38% (44.88 → 35.73 mg GAE/100 g); ANTH germination: −3.11% (0.54 → 0.53 mg CGE/g); ANTH fermentation: −3.23% (0.54 → 0.53 mg CGE/g); ANTH roasting: −5.07% (0.54 → 0.52 mg CGE/g); AOA germination: +81.07% (10.41 → 18.85%); AOA fermentation: +39.28% (10.41 → 14.50%); AOA roasting: −6.53% (10.41 → 9.73%)	[50]
Fermentation and controlled germination	Foxtail millet	Soaking: 12 h at 25 °C (1:15), germination: 48 h at 25 °C in darkness, fermentation: 20 h at 38 °C with lactic acid bacteria to pH 3.0	Dry basis	TPC combined: +98.2% (2.99 → 5.92 mg GAE/g); TFC combined: +16.6% (6.29 → 7.33 mg QE/g); AOA DPPH combined: +81.5% (18.42 → 33.44%); AOA FRAP combined: +33.5% (10.22 → 13.64 μmol TE/g); Reducing power combined: +184.5% (79.63 → 226.58 mg AAE/100g)	[42]

Note. This table summarizes the effect of different biological inducers, mainly microbial fermentation processes and their combinations with germination, applied to cereals and pseudocereals to enhance the accumulation of bioactive compounds. The quantitative results show percentage changes compared to controls without treatment. Abbreviations: AAE, Ascorbic acid equivalents; ABTS, 2,2′-azino-bis(3-ethylbenzothiazoline-6-sulfonic acid); AgNPs, Silver nanoparticles; ANTH, Anthocyanins; AOA, Antioxidant activity; BF, Bioreactor fermentation; *β*-GLU, *β*-Glucan; CAROT, Carotenoids; CE, Catechin equivalents; CGE, Cyanidin-3-glucoside equivalents; CHL-*a*, Chlorophyll *a*; CHL-*b*, Chlorophyll *b*; DPPH, 2,2-diphenyl-1-picrylhydrazyl; FAA, Free amino acids; FLp, Fermented with *L. plantarum*; FL*p*-SG, Fermented with *L. plantarum* after soaking and germination; FPHE, Free phenolic compounds; FRAP, Ferric reducing antioxidant power; FSp, Fermented spontaneously; FYLc, Fermented with yeasts and lactic acid bacteria; GABA, *γ*-Aminobutyric acid; GAE, Gallic acid equivalents; G+F, Germination plus fermentation; GRI, Germination rate index; GVC, Germination velocity coefficient; MGT, Mean germination time; PF, Plate fermentation; PHYA, Phytic acid; QE, Quercetin equivalents; RH, Relative humidity; SSF, Solid-state fermentation; TDC, Total deoxy-anthocyanidins; TE, Trolox equivalents; TFC, Total flavonoid content; TPC, Total phenolic content.

**Table 8:** Combinations of physical, chemical, and biological inducers and their synergistic effects on the accumulation of bioactive compounds during cereal germination.

Combination of Inductors	Reported Synergies	Species Studied	Measurement Basis	Bioactive Compounds and Quantitative Results	**Reported Limitations**	Ref.
US and fermentation with a complex starter culture	Synergy between ultrasound treatment and fermentation with a complex starter culture	Spring soft wheat and spring barley	Not specified	GABA wheat Zauralochka: +370% (2.7 → 12.7 mg/100g); GABA wheat Erythrosperium: +210% (7.1 → 22.0 mg/100g); GABA barley Chelyabinets: +220% (6.5 → 20.8 mg/100g); TFC wheat Zauralochka: +35% (values not specified); TFC wheat Erythrosperium: +45% (values not specified); TFC barley Chelyabinets: +68% (values not specified); AOA wheat Zauralochka: +31% (values not specified); AOA wheat Erythrosperium: +38% (values not specified); AOA barley Chelyabinets: +51% (values not specified)	Germination and fermentation reduce starch content	[3]
Controlled germination, gibberellic acid (GA3), indole-3-acetic acid (IAA), KNO_3_, MgSO_4_, H_2_O_2_, ascorbic acid (ASCA) and H_2_O	GA3 and KNO3 showed synergistic effects for wheat and oats, while GA3 and IAA were more effective for barley	Wheat, barley and oats	Wet basis/Dry basis	Wheat with MgSO_4_: CHL: +35% (3.0 → 4.05 mg/g); Wheat with H_2_O_2_: CHL: +15% (3.4 → 4.2 mg/g); Barley with H_2_O_2_: CHL: +150% (1.0 → 2.5 mg/g); Oats with IAA: CHL: +77% (4.5 → 8.0 mg/g); CAROT in wheat with KNO_3_: +63% (0.24 → 0.39 mg/g); Wheat germination with GA_3_: +12% (81 → 91%)	Variation in response depending on cereal type	[32]
Pretreatments (soaking, ultrasound and alkaline thermo-hydrolysis) + controlled germination	US pretreatment combined with germination shows synergistic effects	Buckwheat and quinoa	Not specified	Buckwheat + ultrasound/germination: TPC: +34% (4.59 → 6.14 mg GAE/g); AOA: +20% (13.29 → 15.95 μmol TE/g); FLVN: +201% (16.33 → 49.21 μg/g); Quinoa + ultrasound/germination: TPC: +8% (2.01 → 2.17 mg GAE/g); AOA: +64% (2.42 → 3.97 μmol TE/g); FLVN: +43% (6.24 → 8.93 μg/g); Buckwheat + thermo-alkaline: TAN: −83% (0.40 → 0.07 mg CE/g); Quinoa + ultrasound: PHYA: −85.5% (10.85 → 1.57 mg/g)	Alkaline treatment decreases TPC	[8]
UV-B radiation and CaCl_2_ supplement	Combined treatment shows synergistic effects for carotenoid enhancement	Yellow corn	Dry basis	LUT: +77.38% (9.15 → 16.23 μg/g); ZEAX: +121.07% (5.60 → 12.38 μg/g); *α*-CRY: +75.19% (1.33 → 2.33 μg/g); *β*-CRY: +65.52% (0.29 → 0.48 μg/g); *α*-CAR: +79.17% (0.24 → 0.43 μg/g); *β*-CAR: +86.49% (0.37 → 0.69 μg/g); SOD: +14.12% (6.02 → 6.87 U/mg protein); POD: +2.66% (9.76 → 10.02 U/mg protein)	UV-B radiation inhibits growth	[69]
US and selenium (selenium enrichment with sodium selenite)	Synergy between ultrasound and low drying temperature (50 °C)	Black rice	Dry basis	GACA: +271% (0.70 → 2.61 mg/g); PROTA: +268% (0.25 → 0.91 mg/g); CYA3GL: +732% (0.24 → 1.97 mg/g); Total phenolics: +146% (1.85 → 4.55 mg/g) with 10 min ultrasound, 50 °C	Limited ultrasound time	[82]
Potassium-enriched biochar (KBC) and gibberellic acid (GA_3_)	GA_3_+KBC synergy for increasing germination, chlorophyll content and reducing antioxidant enzymes under osmotic stress	Wheat	Wet basis/Dry basis	CHL-*a*: +34.35% (1.06 → 1.43 mg/g); CHL-*b*: +20.96% (0.68 → 0.82 mg/g); Total chlorophyll: +29.12% (values not specified); Electrolyte leakage: −11.02% (values not specified) with GA_3_+KBC under low stress	More field-level research is needed	[83]
Hormopriming (GA_3_, IAA), halopriming (KNO_3_, MgSO_4_), osmopriming (H_2_O_2_, ascorbic acid), hydropriming (distilled water)	No combinations of inductors were reported in this study	Wheat	Dry basis	TPC KNO_3_: +16.7% (19.28 → 22.50 mg GAE/g); TFC H_2_O_2_: +5.9% (25.39 → 26.88 mg RUE/g); CATCH H_2_O: +60.3% (6.80 → 10.90 μg/g); CHLA GA_3_: +1008.8% (0.34 → 3.77 μg/g); HYBA GA_3_: +1183.3% (0.06 → 0.77 μg/g); SINA H_2_O: +113.3% (0.83 → 1.77 μg/g); RUTI H_2_O: +158.3% (1.20 → 3.10 μg/g); NARG GA_3_: +214.5% (1.72 → 5.41 μg/g); QUER GA_3_: +282.4% (0.17 → 0.65 μg/g); AOA KNO_3_: +20.8% (51.23 → 61.89%)	Study limited to laboratory conditions	[94]
Controlled germination and thermal treatment (tempering and roasting)	Synergy between germination and thermal treatment for phenolic compounds and antioxidants	Rice	Not specified	TPC green malt roasted (150 °C, 45 min): +113.8% (3.25 → 6.95 mg GAE/g); TPC tempered malt roasted (125 °C, 90 min): +83.1% (3.25 → 5.95 mg GAE/g); Amino acids (50 °C, 60 min): +80% (5.04 → 9.08 mg/g); AOA tempering (60–90 min): +46.21% (25 → 36.04%)	High temperatures (>175 °C) reduce bioactives	[51]
US (US) and PEF	Synergistic effect between US and PEF, resulting in greater bioactive increase	Wheat	Wet basis	TPC US+PEF: +8.59% (305.23 → 331.45 μg GAE/g); TFC US+PEF: +14.06% (178.34 → 203.42 μg CE/g); CHL US+PEF: +12.06% (1.74 → 1.92 mg/100 mL); AOA DPPH US+PEF: +8.58% (1.63 → 1.74 mmol TE/L); AOA ORAC US+PEF: +2.34% (5.12 → 5.24 mmol TE/L)	Long-term stability unknown	[96]
US (US) and microwave (MW)	No specific synergies reported between inductors	Sorghum	Not specified	GABA ultrasound (15 min): +182% (30.89 → 87.14 μg/g); GABA microwave (10% power): +117% (30.89 → 66.97 μg/g); TPC ultrasound (20 min): +18.8% (17.89 → 21.26 mg GAE/100g); AOA ultrasound (10 min): 84.53% vs. 60.61% in control	Prolonged ultrasound times reduce effectiveness	[66]

Note. This table summarizes different combinations of inductors used to enhance the accumulation of bioactive compounds during cereal germination, detailing the reported synergies, studied species, analyzed compounds, and quantitative results. The implementation complexity is classified as low, medium, or high according to technological requirements and process control. All documented combinations were developed and validated at pilot scale, demonstrating their potential for industrial scaling and commercial application. Abbreviations: AOA, Antioxidant activity; ASCA, Ascorbic acid; CAROT, Carotenoids; CATCH, Catechin; CE, Catechin equivalents; CHL, Chlorophyll; CHLA, Chlorogenic acid; CaCl_2_, Calcium chloride; DPPH, 2,2-diphenyl-1-picrylhydrazyl; FLVL, Flavonols; FLVN, Flavonoids; GA3, Gibberellic acid; GABA, *γ*-Aminobutyric acid; GACA, Gallic acid; GAE, Gallic acid equivalents; IAA, Indoleacetic acid; LUT, Lutein; NARG, N-Acetylglutamine; O_3_, Ozone; ORAC, Oxygen radical absorbance capacity; PHYA, Phytic acid; QUER, Quercetin; TE, Trolox equivalents; TFC, Total flavonoid content; TPC, Total phenolic content; ZEAX, Zeaxanthin; *α*-CRY, *α*-Cryptoxanthin.

**Table 9:** Inductors for the enrichment of bioactive compounds in cereals: applications, matrices, characteristics, and commercial applications.

Application	Inductors	Matrices	Benefits and Characteristics	Technological Challenges	Market Opportunities	**Ref.**
Foods with ACE inhibitory activity	Specific inductors (PLP, chitosan)	Barley, buckwheat, rice	High GABA content; ACE inhibitory peptides; phenolic compounds (rutin, catechin) with antihypertensive activity; reduction of factors that promote hypertension. Research development status.	Clinical validation; effective concentrations in final product; stability during processing; optimization of treatment conditions	Functional foods for blood pressure control; preventive products for cardiovascular health; foods targeted at the hypertensive population	[5,17,25,30,112]
Foods with improved mineral bioaccessibility	Germination combined with lactic acid fermentation	Sorghum, millet, corn	Significant reduction of phytates, tannins, oxalates and other antinutrients; increase in bioavailability of iron, zinc and calcium; moderate increase in phenolic compounds. Research/Pilot development status	Prolonged processing time; optimization by cereal type; balance between antinutrient reduction and bioactive preservation	Naturally fortified food products; foods for populations with micronutrient deficiencies, especially women and children in Africa	[4,24,38,77]
Sports foods	US + low-intensity microwave	Oats, rye, corn	High protein content with branched-chain amino acids; bioactive peptides with regenerative properties; GABA for recovery; antioxidants to reduce post-exercise oxidative stress; gradually absorbed carbohydrates; bioavailable minerals. Pilot development status	Standardization; organoleptic profile; formulation for different consumption times; stability	Muscle recovery; sports performance; pre/post-training products; natural alternatives to synthetic supplements	[50,66]
Infant foods	Low-temperature germination (28 °C) + ultrasound	Quinoa, amaranth, millet	Improved essential amino acid profile; increase in B vitamins; reduction of antinutritional factors; increased DHA and essential fatty acids; bioavailable phenolic compounds; higher protein digestibility. Research development status.	Microbiological safety; digestibility; allergenicity; sensory acceptability; stability during processing	Nutritious porridges and snacks for cognitive development; foods for early childhood; premium organic products	[4,11]
Foods for glycemic control	Cold plasma (CAPP and DBD) + germination	Rice, barley, wheat, buckwheat	Modification of starch structure for reduced digestibility; increase in phenolic compounds with *α*-amylase and *α*-glucosidase inhibitory properties; increase in soluble fiber; high GABA content; bioactive compounds with hypoglycemic activity. Research development status.	Specialized plasma equipment; parameter optimization according to cereal; in vivo validation; variable effect depending on variety	Foods for prevention and management of diabetes; low glycemic index products; foods for weight control	[31,70,88,100]
Foods for brain health	Hydrogen-rich water (HRW) + controlled germination	Wheat, rice, buckwheat	Increase in GABA, improvement in enzymatic antioxidant content (SOD, CAT), increase in neuroprotective phenolic compounds, higher bioactive protein content, stimulation of metabolic pathways related to neuroprotection. Research development status.	Stability of dissolved hydrogen; specific equipment; optimization of treatment conditions; partially understood mechanisms of action	Foods for cognitive improvement; products for the prevention of age-related mental deterioration; functional foods for students and professionals	[15,37,108,113]
Functional beverages	Germination + lactic fermentation	Oats, barley, quinoa	High content of *β*-glucans with hypocholesterolemic properties; avenanthramides with anti-inflammatory activity; phenolic compounds and flavonoids with antioxidant capacity; bioactive peptides with ACE inhibitory activity. Commercial limited development status.	Stability during shelf life; processing that preserves bioactives; standardization of content; limited solubility	Products for cardiovascular health; premium plant-based beverage market; athletes and fitness	[4,5,24,50]
GABA-rich functional beverages	Plasma-activated water (PAW) + controlled germination	Barley, rice, wheat	High GABA content; increase in total free amino acids; increase in antioxidant enzymes; higher chlorophyll content; release of phenolic compounds with antioxidant activity; improved sensory profile (reduction of undesirable volatile compounds). Research development status.	Limited shelf life of PAW; industrial scalability; precise control of reactive species; need for specialized equipment	Beverages for stress reduction; products to improve sleep quality; functional foods for hypertension; fermented beverages with probiotic properties	[17,37,45,68]
Sprouts enriched with specific phytonutrients	Moderate abiotic stress (salinity, CaCl_2_, sucrose)	Buckwheat, rice, corn	Specific increase in target compounds: CAROTS, GABA, flavonoids (rutin, quercetin, kaempferol), specific phenolic acids (ferulic, *p*-coumaric); metabolic adaptation that increases antioxidant defense systems. Research development status.	Balance between stress to induce bioactive compounds and acceptable yield; optimization by species; knowledge transfer to commercial scale	Functional foods directed at specific conditions; ingredients with specific health claims; components for specialized supplements	[33,83,87,89]
Functional sprouts for direct consumption	(PEF 3–6 kV·cm^−1^) + cold plasma	Wheat, buckwheat, quinoa	Increased content of chlorophylls, GABA, flavonoids, CAROTS, antioxidant enzymes, significantly elevated antiradical capacity (DPPH, ABTS). Research/pilot development status.	Specialized equipment; precise parameter control; energy cost; variable yield depending on species	Ready-to-eat superfoods; functional living foods; components for smoothies and juices; nutritious vegetable snacks	[17,31,37,47,96]
Enriched breakfast cereals	Germination (malting) + controlled drying	Wheat, oats, barley, quinoa	Increase in total polyphenols, flavonoids, improved antioxidant activity (DPPH, ABTS, FRAP), increased B vitamins, Maillard reaction compounds with antioxidant properties, and improved amino acid profile. Commercial development status.	Balance between thermal processing and preservation of bioactive compounds; development of attractive sensory profiles; standardization of processes	Value-added cereals; products for premium segments; foods with “whole grain plus” claims; alternatives to conventional cereals	[14,51,53,115]
Bioactive concentrates	Specific inductors depending on target compound (MgSO_4_, KNO_3_, H_2_O_2_)	Rice, buckwheat, barley, sorghum	Standardized GABA concentrates for antihypertensive applications; extracts rich in avenanthramides for anti-inflammatory applications; concentrates of specific flavonoids (rutin, quercetin) with high bioavailability; purified bioactive peptides. Laboratory/Pilot development status.	Purification; standardization; stability; scaling up of extraction processes; production cost	Ingredients for nutraceuticals; food supplements; medical foods; specialized food ingredients; techno-functional ingredients	[30,70,76,112]
Germinated seeds with optimized phytochemical profile	Static magnetic fields (1–10 mT)	Rice, buckwheat, wheat, barley	Significant increase in polyphenols, flavonoids, rutin, GABA, alteration of secondary metabolism without negatively affecting germination, and modification of enzymatic activity in key biosynthetic pathways. Research development status.	Equipment for magnetic field generation; optimization of intensity and exposure time; mechanisms of action not fully elucidated	Premium sprouts with specific functional properties; ingredients for food supplements; components for designer foods	[15,103,122]
Germinated seeds with an improved sensory profile	Pretreatment with organic acids (citric, lactic)	Adlay, rice, millet	Moderate increase in total phenols, reduction of undesirable volatile compounds, improved amino acid profile, improved antioxidant activity, optimized texture. Research development status.	pH optimization; balance between sensory profile and bioactive compounds; variability between cereals; microbiological control	Sprouts with better sensory acceptance; products for introduction to the conventional consumer market; sensorially attractive functional foods	[16,38,72,114]
Functional flours	Moderate thermal treatments + SA	Wheat, rice, amaranth	Increase in alkylresorcinols with anticancer activity; total phenolic compounds; increase in GABA; improved soluble dietary fiber; reduction of antinutrients; higher mineral bioavailability. Pilot/Commercial development status.	Maintaining technological properties; treatment homogeneity; quality control during processing; balance between bioactive compound content and functionality	Functional bakery; premium gluten-free market; foods with specialized nutritional value; products for diabetics	[66,124,126]
Germinated flours with improved antioxidant activity	UV-C light (200–280 nm) + controlled germination	Wheat, amaranth, millet	Increased total phenolic compounds; increased total flavonoids; improved antioxidant activity (DPPH); reduction of antinutritional factors; improved essential amino acid profile; structural modification of starch for better functionality. Research development status.	Control of exposure time and distance; optimization of conditions according to cereal; balance between bioactive activity and yield	Ingredients for bakery with functional properties; premium flours for conscious consumers; foods with natural antioxidant claims	[28,50,65,77,80]
Naturally biofortified ingredients	Nanoparticles (ZnO, Fe) + germination	Corn, rice, millet	Significant increase in CAROTS, phenolic compounds, higher bioavailability of essential minerals, improved nutritional profile (amino acids, vitamins), and increased stress resistance. Research development status.	Controlled synthesis of nanoparticles; regulation and consumer acceptance; dose optimization; long-term safety evaluation	Naturally biofortified foods; products to combat nutritional deficiencies in vulnerable populations; alternatives to chemical fortification	[87,91,97,125]
Functional malts	Chemical elicitors (gibberellic acid, SA)	Barley, wheat, sorghum	Increase in enzymatic activity (*α*-amylase, *β*-glucanase) for better functionality; higher content of phenolic compounds and flavonoids; reduction of antinutrients during malting; optimized biochemical profile for specific applications. Pilot development status.	Precise control of hormonal treatments; specific optimization according to variety; balance between enzymatic activity and bioactive compounds	Functional beers; malt extracts as ingredients; specialty malts for craft breweries; ingredients for bakery and pastry	[32,39,44,94,107]
Functional microgreens	Controlled germination with saline stress (NaCl 100–300 mM) + UV-B light	Buckwheat, quinoa, barley	High flavonoid content, superior antioxidant capacity, increased GABA content, unique phytochemical profile with high levels of rutin, catechins and phenolic acids. Significant concentrations of anthocyanins in colored varieties. Research/pilot development status.	Precise control of stress conditions; commercial scalability; reduced shelf life; consistent quality control; batch-to-batch variability	High value-added foods; gourmet market; health-conscious consumers; ingredients for premium culinary applications	[26,36,49,89]

Note. This table presents a synthesis of potential applications for cereals and pseudocereals processed using improved germination techniques and emerging inductors. Abbreviations: ABTS, 2,2-diphenyl-1-picrylhydrazyl; ACE, Angiotensin-converting enzyme; *α*-amylase, Alpha-amylase; *α*-glucosidase, Alpha-glucosidase; *β*-GLU, *β*-Glucan; *β*-glucans, Beta-glucans; CAPP, Cold Atmospheric Pressure Plasma; CAROT, Carotenoids; CAT, Catalase; CaCl_2_, Calcium chloride; DBD, Dielectric barrier discharge; DHA, Docosahexaenoic acid; DPPH, 2,2-diphenyl-1-picrylhydrazyl; FRAP, Ferric reducing antioxidant power; GABA, *γ*-Aminobutyric acid; H_2_O_2_, Hydrogen peroxide; HRW, Hydrogen-rich water; KNO_3_, Potassium nitrate; MgSO_4_, Magnesium sulfate; mT, Millitesla; PAW, Plasma-activated water; PEF, Pulsed electric field; PLP, Pyridoxal phosphate; SOD, Superoxide dismutase; US, Ultrasound.

## Data Availability

No new data were created or analyzed in this study.
